# Natural history of *S*-adenosylmethionine-binding proteins

**DOI:** 10.1186/1472-6807-5-19

**Published:** 2005-10-14

**Authors:** Piotr Z Kozbial, Arcady R Mushegian

**Affiliations:** 1 Stowers Institute for Medical Research, 1000 E. 50th St., Kansas City, MO 64110, USA; 2 Department of Microbiology, Molecular Genetics, and Immunology, University of Kansas Medical Center, Kansas City, Kansas 66160, USA

## Abstract

**Background:**

*S*-adenosylmethionine is a source of diverse chemical groups used in biosynthesis and modification of virtually every class of biomolecules. The most notable reaction requiring *S*-adenosylmethionine, transfer of methyl group, is performed by a large class of enzymes, *S*-adenosylmethionine-dependent methyltransferases, which have been the focus of considerable structure-function studies. Evolutionary trajectories of these enzymes, and especially of other classes of *S*-adenosylmethionine-binding proteins, nevertheless, remain poorly understood. We addressed this issue by computational comparison of sequences and structures of various *S*-adenosylmethionine-binding proteins.

**Results:**

Two widespread folds, Rossmann fold and TIM barrel, have been repeatedly used in evolution for diverse types of *S*-adenosylmethionine conversion. There were also cases of recruitment of other relatively common folds for *S*-adenosylmethionine binding. Several classes of proteins have unique unrelated folds, specialized for just one type of chemistry and unified by the theme of internal domain duplications. In several cases, functional divergence is evident, when evolutionarily related enzymes have changed the mode of binding and the type of chemical transformation of *S*-adenosylmethionine. There are also instances of functional convergence, when biochemically similar processes are performed by drastically different classes of *S*-adenosylmethionine-binding proteins.

Comparison of remote sequence similarities and analysis of phyletic patterns suggests that the last universal common ancestor of cellular life had between 10 and 20 *S*-adenosylmethionine-binding proteins from at least 5 fold classes, providing for *S*-adenosylmethionine formation, polyamine biosynthesis, and methylation of several substrates, including nucleic acids and peptide chain release factor.

**Conclusion:**

We have observed several novel relationships between families that were not known to be related before, and defined 15 large superfamilies of SAM-binding proteins, at least 5 of which may have been represented in the last common ancestor.

## Background

*S*-adenosylmethionine (SAM or AdoMet) is a conjugate of nucleotide adenosine and amino acid methionine, two ubiquitous biological compounds that almost certainly were present in the common ancestor of living cells and may have been found in the prebiotic environment on Earth, predating the origin of Life itself [1]. SAM is an essential metabolic intermediate in every studied cellular life form, and each cellular organism has several SAM-utilizing enzymes. One relatively well-understood biological role of SAM is to donate methyl groups for covalent modification of different substrates – from as simple as oxidized arsenic, chloride, bromide, and iodine ions [2-4], to as complex as rRNA, tRNA, and essential proteins, whose methylation status can serve as a regulatory signal for maturation and control interactions with other macromolecules ([5-7] and references therein).

Methyl transfer is but one of many biochemical processes requiring SAM. Enzymatic reactions that involve interaction of proteins with SAM or its structurally similar derivatives include transfer or methylene, aminoalkyl, ribosyl, and 5'deoxyadenosyl groups; formation of 5'deoxyadenosyl radical, which can be used as a redox intermediate in many reactions; SAM decarboxylation; and *de novo *synthesis of SAM from adenosine and methionine. There are also numerous interactions between SAM and non-enzymatic proteins, where SAM serves as a ligand triggering a regulatory change in the effector protein.

Despite the interest in this amazing variety of functions associated with SAM, and the known three-dimensional structures for representatives of almost every class of SAM-dependent enzymes, the structural, functional, and evolutionary relationships between the SAM-binding domains remain not well understood. Do all or some of the SAM-binding proteins share common evolutionary ancestry? How many distinct structural modes of interaction between SAM and protein are there? Is there strong or weak correlation between conservation of sequence and structure, the mode of SAM binding, and the chemical reaction facilitated by the enzyme? Finally, what may have been the repertoire of SAM-binding proteins in the ancestral organisms – in particular, in LUCA, the Last Universal Common Ancestor of the three present-day domains of Life – Bacteria, Archaea, and Eukarya?

We sought to address these questions by comparing sequences and structures of various groups of SAM-binding domains recognized in proteins. We describe several previously unsuspected relationships between some of such groups, predict novel members for many of them, and conclude that LUCA may have had more than a dozen of SAM-binding proteins, belonging to several distinct folds.

## Results and discussion

We have adopted the iterative comparison strategy, using the known or suspected SAM-binding protein domains as the queries in increasingly sensitive probabilistic methods of sequence modeling and database searching. In many cases, a SAM-binding part of the protein constitutes only part of the polypeptide chain. For example, methyltransferases typically consist of well-conserved SAM-binding portions and highly variable substrate-binding regions, sometimes further supplemented with portable domains also found in otherwise unrelated proteins, such as chromo domain interacting with methylated histone tails in eukaryotes, or PUA domain that probably interacts with RNA [8]. In this work, we are concerned with the protein moieties that bind SAM, so we neither examine these other domains, not consider methyltransferases that utilize other sources of methyl groups, like folate or methylcobalamin derivatives. We did not describe isoprenylcysteine carboxyl methyltransferase (ICMT), an integral endoplasmic reticulum membrane protein with unknown structure [9,10] (reviewed in ref. [11]).

The phylogenetic relationships inside of several recognized groups of SAM-binding proteins, especially within Rossmann-fold SAM-dependent methyltransferases, have been reviewed recently [12,13]. Although we summarize and extend their observations, our main focus is on the analysis of more distant, previously unexamined, relationships.

### Versatile α/β architectures adapted for SAM binding

#### Rossmanoids: ancient and ubiquitous SAM-dependent transferases

The majority of SAM-dependent methyltransferases belong to a large class of enzymes with the Rossmann-like fold, one of the more common arrangements of protein spatial structure, observed in dozens of diverse families of enzymes [14]. SAM-dependent methyltransferases are a large group of enzymes within the Rossmanoid class, and they account for a substantial fraction of all proteins in completely sequenced genomes; for example, with 1.7% of genes in *Helicobacter pylori *J99 coding for known or predicted SAM-dependent methyltransferases, this group makes the list of 10 most commonly used sequence and structure families in that species [15].

**Figure 1:**
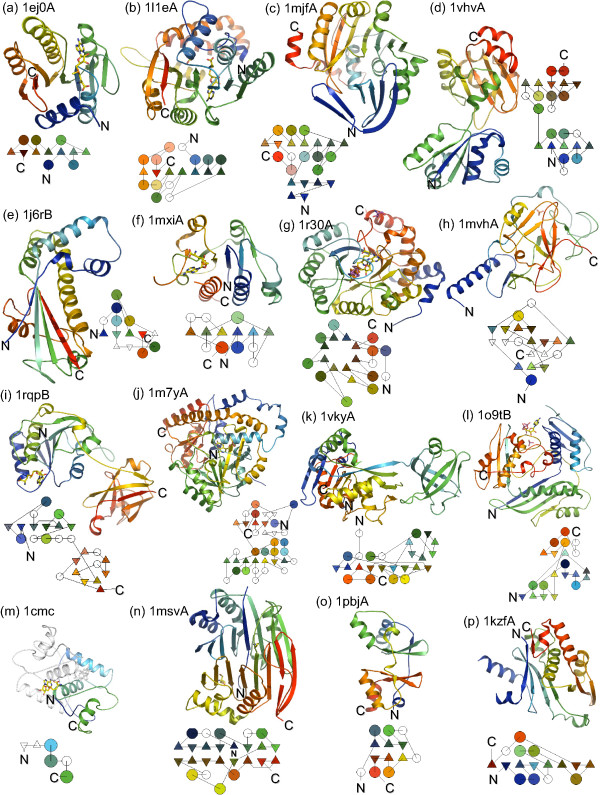
**Fold and topology of SAM binding proteins**. Corresponding fragments of cartoon and topology representations of selected structures were rainbow colored from N-terminal (blue) to C-terminal (red) end. Less significant fragments of secondary structure were left white in topology diagrams. Reference to other representative structures are provided (in parentheses) as SCOP sunid numbers (i.e. ref. [139]). **(a) **1ej0A, Rossmann-fold methyltransferase (53335 excluding: 102555, 69556, 69557, and 69560); **(b) **1l1eA, cyclopropane fatty acid synthase (69560); **(c) **1mjfA, spermidine synthase (69557); **(d) **1vhvA, porphyrin C-methyltransferase (53789); **(e) **1j6rB, Met synthase reactivation domain (methyltransferase; 56506); **(f) **1mxiA, SPOUT methyltransferase (89629, 75218); **(g) **1r30A, SAM dependent radical enzyme (102114); **(h) **1mvhA, SET domain methyltransferase (82199); **(i) **1rqpB, 5'-fluoro-5'-deoxyadenosine synthase (102521, 101851); **(j) **1m7y, 1-aminocyclopropane-1-carboxylate (ACC) synthase (53441, 64130, and similar but different: 53439); **(k) **1vkyA, tRNA-ribosyl transferase-isomerase (111338); **(l) **1o9tB, methionine adenosyltransferase (55972); **(m) **1cmc, MetJ – methionine repressor (dimer; 100972); **(n) **1msvA, SAM decarboxylase (56275); **(o) **1pbjA, CBS domain (dimer; 54630 – not all CBS domains bind SAM); **(p) **1kzfA, acyl-homoserine lactone synthase (75508).

In the most basic arrangement, the Rossmann-like fold consists of alternating β-stranded and α-helical regions, with all strands forming a central relatively planar β-sheet, and helices filling two layers, one on each side of the plane. As with many other Rossmann-like folds, the N-terminal β-strand of methyltransferases is located in the middle of the sheet, and the strand topology is 3214576, with the 7^th ^strand antiparallel to all other strands (Figure 1a). Yet another typical feature of Rossmanoid enzymes is that the functionally important, conserved residues are often located in the C-termini of the β-strands or in the adjoining loops [14]. Some methyltransferases conform to this plan quite well, with an occasional addition of an extra helix or a β-hairpin [16], or, rarely, deletion of one or both of strands 6 and 7 [17]. Most methyltransferases, however, contain additional domains appended or inserted into the basic Rossmann fold [16].

**Figure 2:**
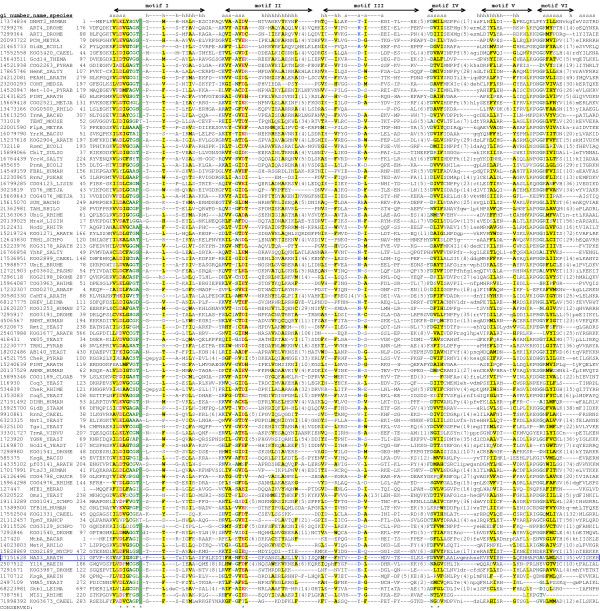
**Multiple sequence alignment of Rossmann-fold methyltransferases and nicotianamine synthase**. Sequences are denoted by NCBI gi number, short protein name (when available, otherwise COG/KOG/Pfam number was used), and abbreviated species name (as in UniProt Knowledgebase [140]). Nicotianamine synthase is marked by a blue box. Conserved motifs are labeled above the alignment. Conserved residues are marked by asterisk. Consensus positions of the secondary structure elements are shown above the alignment. Numbers in parentheses indicate number of residues omitted for clarity. Residues are highlighted according to the amino acid properties. **Gray shading **indicates conservation of single residue. **Red font **indicates conservation of acidic residues (D, E). **Cyan font **indicates conservation of Ser/Thr (S, T). **Blue gray font with yellow shading **indicates conservation of aliphatic residues (I, L, V). **Dark blue font **indicates conservation of basic residues (H, K, and R). **Green font **indicates conservation of tiny residues (A, G, and S). **Blue font with yellow shading **indicates conservation of aromatic residues (F, H, W, and Y). **Pink font **indicates conservation of charged residues (D, E, H, K, and R). **Dark green font **indicates conservation of small residues (A, C, D, G, N, P, S, T, and V). **Bright blue font **indicates conservation of polar residues (C, D, E, H, K, N, Q, R, S, and T). **Blue font with pale yellow shading **indicates conservation of big residues (E, F, H, I, K, L, M, Q, R, W, and Y). **Black font with yellow shading **indicates conservation of hydrophobic residues (A, C, F, G, H, I, L, M, T, V, W, and Y).

Notwithstanding the insertions of additional domains and structural elaborations, comparative sequence analysis of the Rossmann-fold methyltransferases identifies the set of five highly conserved regions of the SAM-binding region, each centered on one or more nearly-invariant residues (Figure 2). They correspond to motifs I-V from motifs initially proposed for DNA:m5C MTases by Posfai et al. [18] (reviewed in refs. [12,19]), but some of the conserved residues highlighted in this work have not been pointed out before (see below). Each motif has a clear counterpart at the structural level. Five motifs are arranged in the same linear order in almost all known methyltransferases, with a notable exception of several groups of DNA- and RNA-methyltransferases, where circular permutation of the sequence results in a main chain fission after motif II, while the spatial structure of the domain and mode of SAM binding remain virtually unperturbed (discussed in more detail by Bujnicki [20]).

The first conserved sequence block (Motif I) includes in its C-terminal part the consensus GxGxG, considered the hallmark SAM-binding site of the Rossmann-fold SAM-dependent methyltransferases. None of three glycine residues is universally conserved, but the replacements are typically by the residues with small side chains, or with propensity of bending the main chain. This agrees with the structure data, indicating that the consensus is located in a loop connecting the first β-strand and the α-helix in the Rossmann fold core. The complete β-strand and part of the preceding loop are also part of Motif I. In the middle of β-strand 1, there is an exceptionally well conserved acidic residue (D or E); one or more conserved positively charged residues are found close to the N-terminus of this strand (Figure 2).

Motif II encompasses β-strand 2 and adjoining turn. A partially conserved acidic residue is common at the C terminus of this strand. Motif III corresponds to β-strand 3, located at the edge of the β-sheet in the Rossmann fold. An acidic residue is partially conserved close to the C-terminus of this strand, too. Whenever the substrate (SAM), its analogs, or reaction product (SAH) are co-crystallized, they are found close to the invariant residues in Motifs I-III (Figure 2 and see below).

Motif IV consists of β-strand 4 and the flanking loops. In this motif again, there is a well-preserved D/E/N residue, located at the extreme N-terminus of the strand, i.e. at the side of the fold that is not involved in substrate binding. Motif V corresponds to the helix following the strand with motif IV. In some Rossmann-fold methyltransferases, it serves as a scaffold for large hydrophobic or aromatic side chains that stabilize the adenine moiety of AdoMet, but it many cases it has been shows these residues are not essential for the MTase activity [21]. Finally, Motif VI corresponds to Strand 5 of the β-sheet, and the preceding tight turn with a nearly-invariant glycine residue.

Several residues from Motifs I-V are known to make direct contact with SAM. In particular, one or more residues in "GxGxG" loop are in contact with the carboxypropyl moiety of SAM, while conserved acidic residue in Motif II forms hydrogen bonds with the ribose hydroxyls (Figure 2; ref. [22]). Variable residues at the C-termini of strand 2 (Motif II) and conserved acidic residue in Strand 3 (Motif III) interact with the nitrous base, while variable residues C-terminal to Strand 4 (Motif IV) appear to contact the amino and sulfonium groups of the methionine moiety of SAM [22]. Residues from motif IV, VI, VIII, and sometimes X are associated with the catalytic pocket, where residues from motif V and VII are important mostly for the structural stability [19,23].

The roles of other conserved residues in SAM-dependent methyltransferases are less well understood. Near-omnipresence of the D/E residue in Motif I suggests that it has an important role. It has been noted [24], that in FtsJ RNA methyltransferase this residue coordinates SAM through a water molecule. In fact, in all 3-D structures of methyltransferases where solvent molecules are present (i.e. PDB structure 1EJ0, 1KYW, and 2ADM), the oxygen atoms in the carboxyl group of this D/E residue make direct contacts with two water molecules, one of which is capable of forming a hydrogen bond with the side chain of methionine moiety of SAM. In some ribose 2'-O-MTases, D/E amino acid conserved in motif I is substituted by tyrosine (Figure 2 and ref. [25]), and it has been proposed that this residue could be used to directly (not via the water molecule) coordinate the amino-carboxyl end of SAM (J.M. Bujnicki, personal communication).

These observations are of interest for understanding the mechanism of methyl transfer by Rossmann-fold methyltransferases. Two best-studied groups of transferases that have Rossmann-like fold and use a nucleotide derivative as a cofactor, namely ATPase-like kinases and nucleoside diphospho-sugar transferases, appear to require a divalent metal cation for polarization of water molecule that can then attack a scissile phosphoester bond [26-29]. Methyltransferases, on the other hand, need to work on a C-S^+ ^bond in SAM, but do not seem to have any metal ion bound in the appropriate position (even though divalent cations have been included in some crystallization media). The proposals for reaction mechanisms of different classes of SAM-dependent methyltransferases include nucleophilic catalysis, with the identity of nucleophile ranging from moderately conserved residues scattered across the SAM-binding domain to bound water molecule [30], as well as S_N_2 reaction, which would require initiation by concerted action of several side chains, or, perhaps, by the amino group of the substrate itself [31]. The highly conserved D/E residue in motif I may, however, provide a unifying theme in the catalysis, by polarizing a water molecule that is close to the methyl group of SAM. The water molecule could either serve as a nucleophile, or aid bond displacement between the sulfonium ion and methyl group in some other way.

Finally, we noticed that the conserved basic residue at the beginning of Motif I and nearly-invariant acidic residue at the beginning of motif IV are typically located within a short distance (3Å or less) of each other, potentially forming a salt bridge that may be important for locking other elements of the Rossmann fold in place (Figure 2).

### Rossmann-fold SAM-binding proteins that do not have methyltransferase activity

#### I. Methylene transferases

Formation of the cyclopropane ring in unsaturated fatty acids by cyclopropane fatty acid synthase [EC: 2.1.1.79] has been studied extensively in bacteria. The reaction involves transfer of a methylene group from SAM to the double bond of an unsaturated acyl chain [13,32].

**Figure 3:**
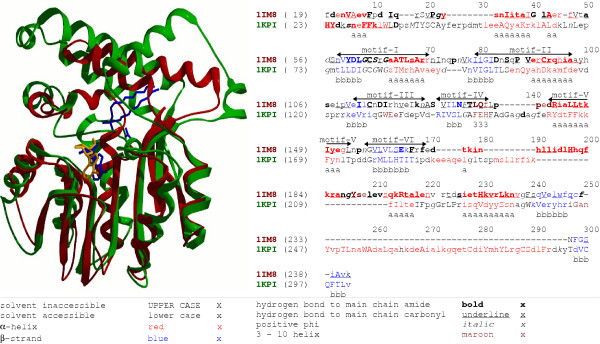
**Structural alignment of cyclopropane fatty acid synthase and Rossmann-fold methyltransferase**. **Red **color – YecO methyltransferase (PDB structure 1IM8:A) **Yellow **color – ligand in YecO methyltransferase. **Green **color – mycolic acid cyclopropane synthase CmaA2 (PDB structure 1KPI:A). **Blue **color – three ligands in CmaA2 (PDB structure 1KPI:A). Motifs I-VI conserved in Rossmann-fold methyltransferases are labeled above the alignment.

Crystal structures of mycolic acid cyclopropane synthases CmaA1, CmaA2, PcaA, and MmaA2 have fold similar to Rossmann-fold methyltransferases (Figure 1b), with the conserved position of SAM and very similar pattern of interactions with the cofactor [32]. A hallmark of methylene transferases is the presence of the carbonate ion (CO_3 _^2-^) at the active center, which probably enables the formation of carbocation intermediate required for completion of the reaction (Figure 3) and ref. [32]. Conserved residues involved in carbonate ion binding (Cys35/Ser, His167/Gln, and Tyr232/Phe – numbered as in PDB structure 1L1E) appear to distinguish methylene transferases from Rossmann-fold methyltransferases.

#### II. Amino alkyl transferases: nicotianamine synthase and spermidine synthase

Nicotianamine synthase (*S*-adenosyl-L-methionine: *S*-adenosyl-L-methionine: *S*-adenosyl-L-methionine 3-amino-3-carboxypropyltransferase, EC: 2.5.1.43) catalyses direct condensation of three molecules of SAM, followed by the formation of an acetidine ring, to yield one molecule of nicotianamine, a chelator of various transition metals ubiquitously present in higher plants. In graminaceous plants, nicotianamine is the precursor of phytosiderophores that are secreted from the roots to solubilize ferric iron in the soil. Reduced levels of endogenous nicotianamine affect the development of plant reproductive organs and seeds' maturation [33].

Protein structure of nicotianamine synthase is not known, but sequence similarity searches indicate a significant similarity between nicotianamine synthase and Rossmann-fold methyltransferases. A PSI-BLAST search, using with *Arabidopsis thaliana *NAS [GenBank:O80483] as a query, matched *Pseudomonas syringae *ubiE/COQ5 methyltransferase [GenBank:YP_233497] with E-value = 1e-21 and score = 105 at 4th iteration; PDBBLAST top match is to (N5)-Glutamine Methyltransferase [PDB:1T43]. Similarity is the highest in conserved motifs II-IV and VI (Figure 2), and motif I is also conserved, in a modified form (F-x-G-S-G-P-x-P). Interestingly, related sequences with the same modification of Motif 1 are found in archaea (*Methanothermobacter thermautotrophicus*, [GenBank:NP_275817], bacteria (*Pseudomonas aeruginosa*, [GenBank:NP_253523], and fungi (*Neurospora crassa*, [GenBank:XP_330777]. The replacement of the conserved D/E/N residue in motif I (see above) may partially explain the change in the functional group that is transferred from SAM: unlike the related Rossmann-fold methyltransferases, nicotianamine synthases lack negatively charged residue in Motif I, so the aminoalkyl moiety of SAM is not bridged to the enzyme by a water molecule and is free to leave in the course of the reaction.

Spermidine synthase (EC: 2.5.1.16) catalyzes the transfer of the aminopropyl group from decarboxylated SAM to putrescine to form spermidine. Putrescine, spermidine and spermine, formed from spermidine, are polyamines essential for the regulation of cell proliferation and differentiation in most species, and gram-negative bacteria outer membrane permeability in response to the acid stress [34,35]. Spermidine synthase is an oligomeric enzyme, each monomer consisting of a C-terminal domain with a Rossmann-like fold and an N-terminal tetramerization β-stranded domain [36].

Spermidine synthase has very high sequence similarity (approx. 70% identity) to putrescine N-methyltransferase. It has been shown that aminopropyl moiety of spermidine synthase inhibitor – AdoDATO (a compound containing both substrate and product moieties) binds in a similar orientation to the homologous part of SAM in Rossmann-fold methyltransferases. However, the binding site in spermidine synthase contains invariant residue Asp101 (PDB structure 1JQ3), located in the middle of glycine-rich loop (corresponding to motif I of Rossmann-fold methyltransferases) making binding cavity too small to accommodate the carboxyl group of SAM, that prevents SAM binding and enables specific binding of decarboxylated SAM [36]. The polyamine moiety of AdoDATO is oriented toward putrescine binding cleft. Invariant Asp170 (corresponding to D/N residue located at the end of β-sheet in motif-IV of Rossmann-fold methyltransferases) is most likely candidate to deprotonate putrescine, enabling it to perform a nucleophilic attack on methylene carbon of decarboxylated SAM [36].

#### III. Acalcynomycin-10-hydroxylase

Aclacinomycin 10-hydroxylase RdmB is a methyltransferase homolog that catalyses a SAM-dependent hydroxylation of the C-10 carbon atom of 15-demethoxy-ε-rhodomycin, a step in the biosynthesis of the polyketide antibiotic β-rhodomycin. In order to activate molecular oxygen, the enzyme uses SAM instead of cofactors usually associated with hydroxylase activity, such as flavins, 2-oxoglutarate, or metal ions. It has been proposed that positive charge of the SAM cofactor plays a role in delocalisation of electrons into the anthraquinone core of the substrate [37]. The C-terminal domain of RdmB has an α/β Rossmann-like fold, and contains the conserved signature DLGGGxG in motif I.

The enzyme lacks methyltransferase activity due to the positioning of SAM in which the methyl group points in a direction unfavorable for a S_N_2 type methyl transfer to the substrate [37]. The difference in SAM-substrate positioning is not well understood, but might be related to differential interactions between SAM binding C-terminal domain and substrate binding N-terminal domain or differences in the conserved loop (amino acids 292–298) [37].

### Non-catalytic Rossmannoids

The lack of detectable SAM binding motifs in several Rossmann-fold methyltransferases suggests that they may be recruited for a new function. For example, the three-dimensional structure of sc-mtTFB (*Saccharomyces cerevisiae *mitochondrial transcription factor B) bears strong resemblance to ribosomal RNA adenine dimethylases (i.e. KsgA and ErmC'). However, several residues required for interaction with SAM are not conserved in sc-mtTFB; in particular, the glycine-rich loop (motif I) contains bulky Tyr residue, and motif IV is poorly conserved.

Human co-orthologs of sc-mtTFB (h-mtTFB1 and h-mtTFB2) have rRNA N6-adenine methyltransferase activity (in an *Escherichia. coli *assay), but mutational analysis of h-mtTFB1 indicates that this activity is not required for transcriptional activation [38].

In Gcd10p/Gcd14p complex – tRNA(1-methyladenosine) methyltransferase of *S. cerevisiae*, the lack of SAM binding was observed in Gcd10p that directs binding of tRNA, where Gcd14p binds the required cofactor *S*-adenosylmethionine [39,40].

In another case, Kar4p (pheromone induced, karyogamy-specific transcription factor) does not bind SAM, where similar (circularly permuted) Ime4p is SAM-binding methyltransferase [41].

In bacterial rRNA:m2G methyltransferases RsmC and RsmD the inactivated domain and the catalytic domain are fused together in one polypeptide [42].

The *de novo *methyltransferase-like protein, DNMT3L, is required for methylation of imprinted genes in germ cells. Although enzymatically inactive, human DNMT3L accelerates DNA and SAM binding to *de novo *DNA methyltransferases [43-45].

### Rossmann-like domain of bacterial fluorinating enzyme

Actinomycete *Streptomyces cattleya *is able to produce C-F bonds using inorganic fluoride. The fluorinating activity requires SAM, and the primary product of the reaction is SAM derivative, 5'-fluoro-5'-deoxyadenosine [46]. The protein, 5'-fluoro-5'-deoxyadenosine synthase FlA, belongs to a conserved sequence family represented in most archaea and in a subset of bacteria [46].

The structure of FlA consists of two domains – a larger N-terminal domain with α/β fold, and a smaller C-terminal β-barrel. Both domains interact with SAM and with reaction products [46]. FlA is a hexamer in solution and trimer in crystal, and three SAM molecules are bound by a trimer, between the N-terminal domain of one subunit and the C-terminal domain of the adjoining subunit. This arrangement, however, appears to be dependent on a long (24 amino acids) loop in the N-terminal domain, which is missing from the closely related sequences in all other species. On the other hand, the linker connecting two domains in a monomer is long enough to allow significant domain motions, and it is plausible that two domains may interact in other oligomeric arrangements and perhaps even within a monomer. Therefore, we speculate that SAM binding by FlA-like proteins from other species may occur in the crevice formed by the N- and C-terminal domains of the same molecule, and the following discussion does not consider the oligomerization state.

The N-terminal domain makes contacts mostly with methionine, ribose, and fluoride ion, and C-terminal domain contacts methionine and adenine ring. The α/β N-terminal domain exhibits several features similar to other enzymatic domains with Rossmann-like topology, namely: three-layer α/β/α architecture; the planar central, mostly parallel β-sheet filling "inside-out" (strand topology 2135467), and concentration of the substrate-binding and catalytic residues in the loops following the C-termini of strands. More specifically, the loop after strand 1 contains Asp16 (numbered as in PDB structure 1RQP) hydrogen-bonded to both hydroxyls of ribose, Leu17 that may be involved in water-mediated interaction with methionine amino group, Asp21 and Ser23, both of which can form hydrogen bonds with the same amino group. Loop following strand 2 contains Trp50 that is able to contact one ribose hydroxyl and perhaps to have Van der Waals interactions with the adenine ring. Loop between strands 3 and 4 contain two ribose interactors, Thr76 and Tyr77. Loop after strand 6 hosts Thr155, which is part of hydrogen-bond network linking two domains via two water molecules and methionine carboxyl group, as well as catalytic Ser158 that is expected to make two polar contacts with deprotonated fluoride ion [46]. Although most of these interactions are provided by residues located in loops at the edge of β-sheet, there is no specific sequence similarity between Rossmann-like domain in fluorination enzyme and in Rossmann-fold methyltransferases. There is also no similarity to other SAM-utilizing enzymes.

### Rossmann-like fold in SPOUT methyltransferases?

A distinct superfamily of SAM-dependent methyltransferases, SPOUT, which includes families specified by bacterial SpoU, TrmD, and TrmH, proteins, as well as many uncharacterized proteins in all three domains of Life, have been shown to share a set of conserved sequence elements and an α/β-type fold [47]. Trm10, a recently characterized tRNA m^1^G_9 _methyltransferase, is also predicted to have this fold [48,49]. All experimentally characterized members of this large superfamily are DNA or RNA methyltransferases. A unique structural feature of this α/β fold is a trefoil knot of two crossing loops in the C-terminal region [47].

Several hallmarks of Rossmann-like structure are evident in the SPOUT fold. There are three main layers, with a central β-sheet sandwiched between two helical layers; the β-sheet is formed "inside-out", with the first and one of the last strands in the center of the sheet; and the SAM ligand interacts mostly with the conserved residues located in the loops at the C-termini of β-strands [50]. There is, however, no sequence similarity between SPOUT-fold methyltransferase and any other Rossmann-fold SAM-binding protein.

Structural similarity between SPOUT-fold and Rossmann-fold methyltransferases (i.e. DALI Z-score = 3.1 for 88 aa with RMSD = 3.7 and sequence identity = 17% for PDB structures 1QAO and 1MXI) is confined mainly to the N-terminal half of those folds. There is no similarity in the C-terminal part, where strands 4 and 5 are rearranged.

### SAM binding inside and outside of β-barrels

#### SAM-radical enzymes: recruitment of ancient enzymatic TIM barrel

A (β/α)_8 _fold, also known as triose phosphate isomerase (TIM)-like barrel, is one of the largest classes of protein structures, exceeding even Rossmann-like fold in omnipresence and versatility [51]. Most of TIM-barrel proteins are enzymes, belonging to almost all of the major EC classes [52]. A well-known version of a TIM barrel is a (β/α)_6 _"semi-barrel," in which the inner layer of slanted β-strands does not form a complete cylinder, but has a lateral opening (PDB structure 1OLT).

Recently, several structures of proteins from a large sequence family of "SAM radical" enzymes (ref. [53]; Figure 1g) have been determined, and it became evident that members of this family have (β/α)_6 _and (β/α)_8 _folds. SAM-radical enzymes utilize non-covalently linked Fe-S cluster and a SAM molecule, in a reductive cleavage reaction that produces methionine and 5'-deoxyadenosyl radical, that can be used to generate further glycyl or thiyl radicals on the same protein molecule or on a coupled enzyme [54]. It has been noted that SAM-radical sequence family is very large, diverse, but can be recognized by a hallmark CxxxCxxC signature close to the N-terminus, followed by another conserved "GG" motif [55].

**Figure 4:**
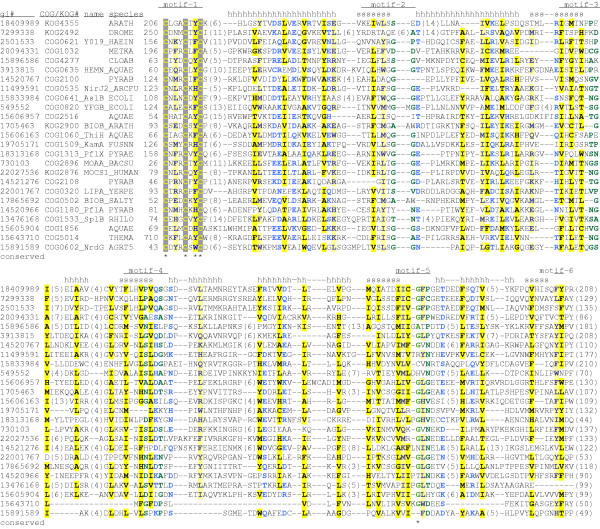
**Multiple sequence alignment of SAM-dependent radical enzymes**. Sequences are denoted by NCBI gi number, conserved domain name (as in NCBI CDD database [141]), short protein name (if available), and abbreviated species name. Secondary structure elements extracted from PDB structure 1OLT coordinates are shown above the alignment. Residues are highlighted according to the amino acid properties with designations as in Figure 2.

We detected more than 2000 non-redundant sequences from SAM-radical family in the sequence databases. Interestingly, when the region containing the three characteristic cysteines was deleted from the queries, the searches resulted in almost the same collection of sequences as with full-length domain, indicating strong evolutionary signal along the stretch of 200–250 residues to the C-terminal side of the CxxxCxxC signature. Multiple alignment of many representative sequences identified four regions with high sequence similarity and three weaker conserved motifs (Figure 4). Comparison of the alignment with the known structures of biotin synthase (PDB structure 1R30), coproporphyrinogen III oxidase (PDB structure 1OLT) and molybdenum cofactor biosynthesis enzyme MoaA (PDB structure 1TV7) suggests structural and functional correlates for these regions and for the most conserved residues within them. The best-conserved motifs correspond to the β-strands of the inner barrel and their C-terminal loops, while the regions of additional partial conservation correspond to the outer-shell α-helices.

Motif 1 includes the most N-terminal β-strand in the (β/α)_6 _barrel (strand 1). Three invariant cysteine residues in the adjoining loop (Figure 4) coordinate the [Fe-S]_4 _cluster, which is present in a similar configuration in all protein structures resolved thus far. One iron atom has no contact with the cysteine side chains, and is instead ligated by the N and O atoms from the amino- and carboxy groups of SAM. Also highly conserved is aromatic or heterocyclic residue (Y, F, or H) preceding the last of the three cysteines; the main chain of this residue seems to form a hydrogen bond with the adenosyl moiety of SAM, but the significance of the side chain conservation is unclear; perhaps it contributes to the non-polar milieu of the bound Fe-S cofactor, preserving it from oxidation.

The second prominent motif does not contain any invariant amino acids, but includes several residues with small side chains, most often two or three glycines in a row (Figure 4). This motif corresponds to the second strand in the barrel and the tight turn after the strand. The main chain of this turn is within a contact distance from the amino group of the methionine part of SAM. The third motif also corresponds to the strand-turn structure. A signature T/S-N-G that follows strand 3 is well conserved; as a rule, residues in this turn form hydrogen bonds with the carboxyl group of methionine in SAM. The fourth motif consists of strand 4 and the loop with a highly conserved acidic or amide residue (D, E, N, or Q). Typically, this residue is within hydrogen-bonding range from both 2'- and 3'hydroxyl groups of the ribose ring of SAM. The strands 5 and 6 followed by loops provide one or more residues that form hydrogen bonds with the amino group of adenosyl; however, sequence conservation in these regions is moderate.

The heterogeneity of the SAM radical protein superfamily is most pronounced in their C-terminal regions, which are responsible for binding of substrates and auxiliary cofactors. On the other hand, the structure and sequence of the N-terminal, SAM-binding region of SAM-radical proteins is well conserved, analogously to the Rossmann-fold methyltransferases. The SAM-binding region is essentially an incomplete (β/α)_6 _"semi-barrel," which is typically modified by evolutionarily diverse elements (commonly consisting of α-helices, but sometimes also containing β-hairpins or small sheets) that serve substrate-binding and regulatory roles.

Rossmann folds and TIM-barrels in fact have quite similar β/α architectures. This becomes especially evident in the case of incomplete barrels. The primary difference is lack of one α-layer in TIM-barrels, and correlated changes in sheet curvature and strand orientation. The two classes of SAM-binding enzymes both use loops between strands and helices to interact with various moieties of relatively extended SAM molecules, but the details of this interaction are quite different (see below).

#### TIM barrel-like catalytic domain in QueA?

Queuosine is a hyper-modified nucleoside in bacterial and eukaryotic tRNAs, produced by a multi-step enzymatic pathway that includes a transfer, with simultaneous isomerization, of ribose moiety from SAM to a modified base in tRNA, called 7-(aminomethyl)-7-deazaguanosine, or preQ1. This step is performed by QueA protein, an *S*-adenosylmethionine:tRNA ribosyltransferase-isomerase. QueA homologs are found in most bacteria, but their sequence is not strongly similar to any other protein family, and high-resolution structure of QueA in complex with SAM is unavailable. We interrogated the fold recognition meta-servers with individual QueA sequences and with a probabilistic model of aligned QueA homologs. The highest 3D-Jury consensus score (69 units, indicating the upper level of the "gray zone" of provocative, if statistically insignificant, sequence similarities [56]) was to pyruvate kinases, a distinct class of proteins with three structural domains. The C-terminal, regulatory domain of pyruvate kinases has no counterpart in QueA. The other two domains are arranged in such a way that a smaller, β-barrel domain is inserted into the larger, α/β TIM-barrel domain but folds independently. Similar arrangement of two domains is predicted for QueA.

When this manuscript was under preparation, the structure of QueA from *Thermotoga maritima *was resolved (PDB structure 1VKY). In agreement with the fold recognition data, it shows an α/β domain with insertion of independently folding β-barrel (Figure 1k). The structure of the α/β domain shows one β-sheet, with preponderance of α-helices on one side (Figure 1k). This structure resembles a semi-barrel, given a strongly curved β-sheet, relative absence of α-helices on the concave side, and a lid-like irregular arrangement of elements that covers the cavity. There is an unresolved protein segment of 32 residues, which should be located close to the inner β-layer of the semi-barrel, and may in fact extend its wall. An unresolved ligand is placed in the proximity of the C-termini of several β-sheets, and if this is in fact SAM, its binding mode would be similar to what is observed in other SAM-binding proteins (see below).

#### β-barrels in QueA and fluorination enzyme

Both QueA and fluorination enzyme structures show fusions of a larger α/β domain and a smaller all-β domain with barrel-like topology. The role of all-β domain in QueA is unclear, but it is not very likely to be involved in interaction with SAM. In contrast, the β-barrel domain in FlA (which, in fact, is more similar to "smashed β-can," with one side caved in, producing a double-concave surface) makes many contacts with the ligand. Proceeding from the N- to C-terminus, the Asp210-His211 (as in PDB structure 1RQP) dipeptide in the loop after the first strand bonds with the amino group of methionine; Asn215 bonds with the amino group of adenine; Ser269 and Arg270 after strand 5 can form 4 hydrogen bonds altogether, all with the carboxyl group of methionine; and at the C-terminus of strand 5, Arg277 and Ala279 provide additional interactions with adenine. While the catalytic mechanism of FlA is dependent on correct positioning of the fluoride atom with regards to ribose, which is mediated by Ser158 in the Rossmann-like domain (see above and reference [46]), the β-barrel domain appears to be essential for correct orientation of SAM, which serves as fluoride acceptor.

### Between the sheets: double-β SAM-binding folds with a common theme of internal domain duplication

#### Decarboxylase

*S*-adenosylmethionine decarboxylase (EC: 4.1.1.50, SAMDC) is a key enzyme in spermidine and spermine biosynthesis. It produces decarboxylated SAM (dcSAM), which then donates aminopropyl group to putrescine or spermidine, two essential intermediates in polyamine biosynthesis. Because polyamines link diverse pathways in cellular metabolic networks, and because chemical inhibitors of SAMDC display potent antitumor and anti-parasite activities [57,58], structure-function relationships of SAMDC are of considerable interest.

SAMDC activities have been purified from all three domains of Life (bacterium *E. coli*, archaea *Methanococcus jannaschii*, and several eukaryotes), and certain common features of the enzymes have been noticed. All three enzymes are processed *in vivo*, forming a small subunit derived from the N-terminus and a large subunit accounting for the rest of the molecule; the N-termini of all large subunits contain a pyruvoyl group, produced from a serine residue by autoprocessing and required for the formation of the Schiff base during catalysis. All studied enzymes form multimers from the heterodimers of large and small subunits. There are also differences among bacterial, archaeal and eukaryotic SAMDC: the length of the precursor proteins in different species varies from 105 to 460 amino acids; mammalian enzymes require putrescine for full activity, *E. coli *enzyme requires Mg^2+ ^cation, while archaeal and plant enzymes apparently do not require those factors. The bacterial enzyme is a tetramer of heterodimers, while eukaryal and archaeal enzymes are homodimerized heterodimers.

**Figure 5:**
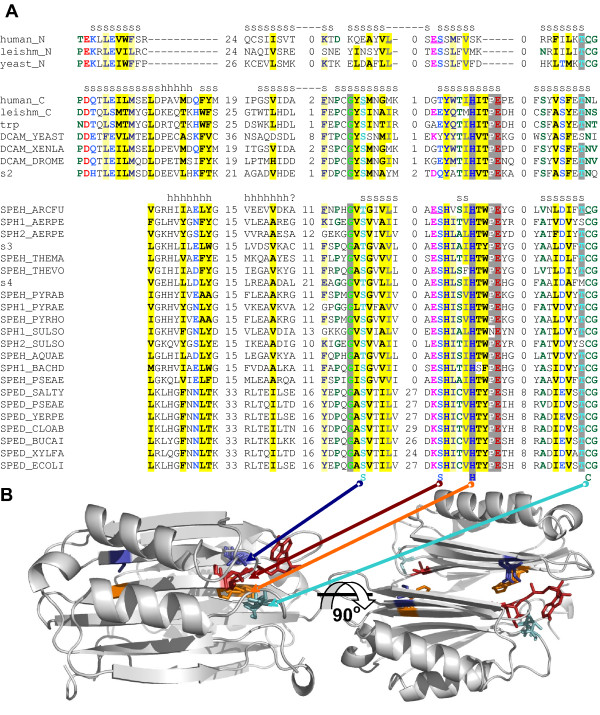
**Multiple sequence alignment of SAM decarboxylases**. Sequences are denoted by gene and species name. **A: **Decarboxylase alignment – the residues underlined on the top line are those involved in SAM-binding, enzyme self-processing, and catalysis. Residues are highlighted according to the amino acid properties with designations as in Figure 2. **B: **Superimposition of the conserved residues in SAMdc from *T. maritima *(PDB structure 1TLU), human (PDB structure 1I7B) and potato (PDB structure 1MHM) are shown on cartoon representation of *T. maritima *structure (gray). **Blue **– Ser; **Red **– Ser and Ser converted into pyruvoyl group (or pyruvoyl group with covalently bound *S*-adenosylmethionine methyl ester); **Orange **– His; **Cyan **– Cys.

Comparative sequence analysis has revealed statistically significant sequence similarity between archaeal and bacterial SAMDC [59]. Multiple alignment of these two classes of SAMDC spans the complete length of the shorter (ca. 120 aa) archaeal enzymes, and also suggests that there are two types of bacterial enzymes – some are about the same size as archaeal SAMDC, and some are longer and phylogenetically distinct (reference [60], Figure 5a, and unpublished observations). No sequence similarity has been reported between these enzymes and eukaryotic SAMDC.

High-resolution structures of eukaryotic SAMDC from humans and plants in complex with substrate analogs and various inhibitors have been reported. The heterodimer folds as a sandwich of two β-sheets between α-helical regions, where the smaller subunit forms a half of one β-sheet, and the larger subunit completes this sheet and accounts for all the strands in the other sheet. The arrangement is unique among the known protein folds, but visual inspection and superposition of the two α-β halves of the molecule revealed their remarkable similarity and suggested the hypothesis of internal duplication [61,62]. The evolutionary origin and catalytic mechanism of prokaryotic SAMDC remained unclear.

Searches of sequence databases with the PSI-BLAST program and more involved probabilistic models of aligned SAMDC enzymes confirmed statistically significant sequence similarity between archaeal and bacterial enzymes, and also, intriguingly, produced several statistically insignificant local matches to one-half of eukaryotic SAMDC sandwich, in the area corresponding to the β-strand 12 in the three-dimensional structure. This strand is positioned next to the active center of the enzyme, and contains residues important for catalysis and/or binding of the substrate (see below). Because both these residues appeared to be preserved in the BLAST output, we sought better statistical validation of this similarity using Metaserver [56]. When SAMDC homolog from archaea *Archaeoglobus fulgidus *was used as a query, the highest 3D-Jury consensus score (46–60) was reported to the set of the eukaryotic SAMDC structures; this score is at the top of the zone with borderline significance, where most of the non-trivial similarities are discovered [56]. The first false positive (bacterial luciferase) was associated with the sharp drop in the 3D-Jury scores (14.5).

Almost complete archaeal sequence can be aligned to the half of eukaryotic template, with just one short gap. Conversely, the aligned region of the template corresponds almost precisely to the C-terminal half of the double sandwich. We conclude that the archaeal enzyme may resemble a half of the eukaryotic SAMDC fold and may be directly related to the pre-duplication ancestor of that fold. Multiple sequence alignment of archaeal, bacterial, and eukaryotic enzymes strongly reinforces these observations (Figure 5a). The C-terminal halves of eukaryotic enzymes could be aligned to prokaryotic homologs directly and unequivocally; the structurally similar N-terminal halves had to be superimposed using the knowledge of secondary structure and information about a few conserved residues.

The functional and evolutionary implications of the alignment are provocative. In mammalian enzymes, SAM decarboxylase is active as a dimer in which each protomer contains one large and one small subunit, and each of the two halves of the sandwich contributes several residues to binding the substrate and actually performing the catalysis. In particular, Ser residue in β-strand 4 of the eukaryotic enzymes, which is converted into catalytic pyruvoyl group, appears to be within a short distance of the carboxyl group of SAM forming a Schiff base adduct with it. Before product release, carbon of decarboxylated SAM is protonated by adjacent Cys (Figure 5b). This protonation regenerate the pyruvoyl group [63]. Also close to the active site is the side chain of the histidine residue in strand 12, which is believed to be responsible for abstraction of a proton from the α-carbon of the catalytic serine during proenzyme processing [64].

Two acidic residues contribute to binding of SAM: glutamic acid at the C-terminus of strand 3 contacts the base, and another glutamate, at the C-terminus of strand 12, interacts with both hydroxyl groups of the ribose ring. All these residues are conserved in eukaryotic SAMDCs – some in the N-terminal half of the sandwich, and others in the structurally equivalent C-terminal half. Interestingly, in archaeal and most bacterial enzymes, the pattern of conservation of these residues appears to be the union of conserved elements in the two halves of eukaryotic enzymes (Figure 5a), as if the bacto-archaeal enzyme is a homolog of one half of the eukaryotic enzyme, and the β-sandwich in the holoenzyme are made of two identical molecules.

When this manuscript was in preparation, the structure of ligand-free holoenzyme from bacterium *T. maritima *was deposited in the database (PDB structure 1TMI). Analysis of this structure confirms this sequence-based prediction and suggests that the bacto-archaeal form is ancestral, and the eukaryotic form has been derived from it by domain duplication/fusion, followed by functional specialization of two halves (most notably, by mutating the C-half of the enzyme so that it no longer undergoes autoproteolysis – Figure 5b).

#### SET domain

Discovered as conserved domain shared by chromatin remodeling proteins Su(var)3–9, E(Z) (short for Enhancer of Zeste) and Trithorax, SET domains turned out to be a distinct class of SAM-dependent methyltransferases. All studied SET methyltransferases transfer methyl group to lysine within various nuclear proteins involved in chromatin function and regulation of transcription, such as histones, TAF10, tumor suppressor p53, but also in such diverse proteins as Rubisco and cytochrome C [65-68].

In SET-domain methyltransferases amine of the substrate lysine residue access the methyl donor (SAM) through a narrow channel connecting the substrate and SAM binding surfaces [69]. SAM binding site and the catalytic center of all studied SET domains seem to be constructed on the unusual but conserved, all-β, knot-like structure [70]. Adenosyl moiety of SAM interacts directly and indirectly, through water, with conserved histidine (PDB structure: 1O9S-His297; 1P0Y-His243). This histidine may serve as a proton acceptor for the hydroxyl group of invariant Tyr (PDB structure 1O9S-Tyr335). The -OH of this Tyr is within 4 Å of the presumptive location of the substrate Lys Nζ, and may be involved in Lys side chain deprotonation (deprotonated Lys is presumed to make a nucleophilic attack on the SAM methyl group). Positively charged amino nitrogen from SAM hydrogen bonds with the side chain of invariant asparagine (PDB structure 1O9S-Asn296). This interaction may contribute to the compact conformation of the SAM molecule.

Phylogenetic analysis of the SET domain suggests that it is an evolutionary innovation in the eukaryotic lineage (with secondary lateral transfer to bacteria, archaea and viruses) [71]. SET domains (Figure 1h and SCOP superfamily: 82199) have a fold unique for SAM binding proteins – a substrate binding subdomain between two structural repeats, which may have evolved by duplication of 3-stranded unit with a generic ligand binding role [71,72]. Those repeats have a β-clip fold formed by double-stranded ribbons sharply bent in two places; the ribbon ends form incomplete barrel.

Similar duplication of a basic three-stranded unit containing the β-clip structural motif probably occurred also in related SAF and dUTPase superfamilies [72], which, however, tend to bind sugar and sugar derivatives [72]. There have been several other cases of adaptation of a generic ligand binding domain for SAM-binding, both in enzymes and in regulatory proteins without catalytic activity (see below).

#### SAM synthetase

**Figure 6:**
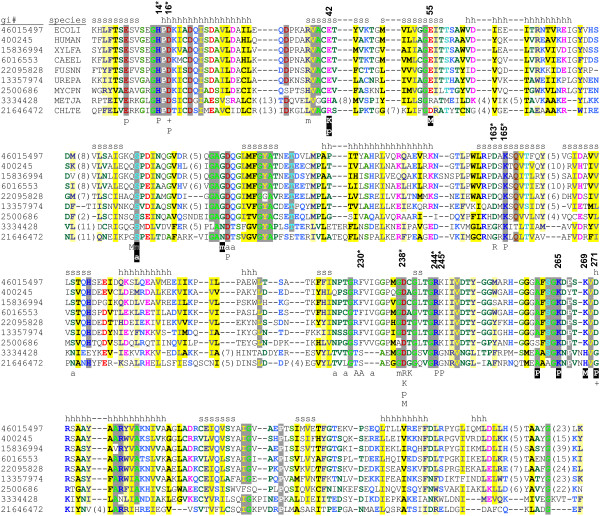
**Multiple sequence alignment of SAM synthetases**. Essential amino acids for the first (*) and the second substrate binding subunit are numbered as in gi:46015497 (PDB structure 1P7L and 1RG9). Aligned sequences represent protein from Archaea (gi:3334428 – *M. jannaschii*), Eukaryota (gi:400245 – *H. sapiens*, gi:6016553 – *C. elegans*), and Bacteria (gi:46015497 – *E. coli*, gi:15836994 – *X. fastidiosa*, gi:22095828 – *F. nucleatum*, gi:13357974 – *U*. *parvum*, gi:2500686 – *M. pneumoniae*, gi:21646472 – *C. tepidum*). Residues are highlighted according to the amino acid properties with designations as in Figure 2. Substrate binding is annotated below the alignment as follows: **small letters **– water mediated interactions; **inverted colors **– interactions with ligand from the second subunit of the homodimeric protein ; **A/a **– adenosyl moiety; **R **– ribosyl moiety, **M/m **– methionine moiety; (**+**) – Mg^2+^; **K **– K^+^; **P **– PPNP/Phosphate moiety. Consensus positions of the secondary structure elements are shown above the alignment. Numbers in parentheses indicate number of residues omitted for clarity.

*S*-adenosylmethionine synthetase (SAM synthetase, ATP:L-methionine *S*-adenosyltransferase, or MAT, EC: 2.5.1.6) is the main, or, possibly, the only enzyme of *de novo *SAM biosynthesis. SAM synthases from bacteria and eukaryotes are closely related at the sequence level and have very similar structures [73]. SAM synthases transiently interact with SAM prior to its release. The mechanism of reaction is thought to rely on conserved His14 (Figure 6), which acts as an acid to cleave the C5'-O5' bond of ATP, while simultaneously a change in the ribose ring conformation from C4'-*exo *to C3'-*endo *occurs, and the S of Met makes a nucleophilic attack on the C5' to form SAM [74].

The fold of bacto-eukaryal SAM synthetase is unique; each protein chain is based on a β-α-β-β-α-β module that folds into a wedge-like shape. A polypeptide chain consists of three such tandemly repeated modules, so that the complete SAM synthetase fold looks like a three-slice cream pie with topping made of β-sheets (Figure 1L). The active form of the enzyme appears to consist of two pies, with β-layers facing each other. Two SAM molecules are bound between the sheets of this dimer. In the *E. coli *enzyme, both subunits contribute many residues to SAM binding (Komoto et al. [74], Figure 6, and PDB structure: 1P7L and 1RG9) In particular, adenosine binds to Asp163, Arg229, Phe230 on one subunit and to Ser99 on another subunit, and interacts with many additional amino acids on both subunits via water-mediated hydrogen bond network. Methionine binds to Gln98 and Asp238 on one subunit, to Glu55 on another, and likewise makes many additional water molecule-mediated contacts. Bacto-eukaryal SAM synthetase is an evolutionary unique sequence and structural family. Even SAM decarboxylase, which is superficially similar in that it also sandwiches SAM between two β-sheets, has no detectable sequence or structure similarity to SAM synthetase.

SAM synthetases from Archaea have been isolated on the basis of their biochemical activity [75]. We performed sensitive searches of the conserved domain database, and found clear evidence for common ancestry of all SAM synthetases (Table 2). Multiple sequence alignment and secondary structure prediction indicate that archaeal enzymes share the same three-dimensional structure as their eukaryotic and bacterial homologs. All known SAM synthetases have conserved GHPD signature containing the main catalytic residue (His14 in *E. coli *gi:46015497) [75]. Despite high sequence divergence between archaeal and bacto-eukaryal enzymes, the complement of substrate binding residues is well-preserved (Figure 6). The apparent common origin of this unique enzyme in all major divisions of Life is of great interest for reconstruction of the repertoire of SAM-binding protein in the ancestral life forms (see below).

**Table 1:** Classes of SAM binding proteins

**Reaction summary**	**Trivial name, EC number**	**Sequence families and spatial folds (summary)**	**Evolutionary roots and status in LUCA**
Methyl transfer	SAM-dependent methyltransferase, EC:2.1.1.-	Five classes: I, Rossmann fold; II, reactivation domain of methionine synthase; III, "corrinoid-like" MTases; IV, SPOUT domain; V, SET domain. Classes I-IV are α/β folds, class V is a β-clip. The Rossmann-fold MTases are the largest class of SAM-dependent enzymes. The folds of Classes II and III are unique. Trm10 and TrmH families of RNA MTases appear to be the modified versions, of, respectively, class IV fold [47], [49] and class I fold fused to PP-superfamily ATPase (this study). GTP MTase of Sindbis-like viruses may belong to α/β class, but specific fold prediction is unavailable	Several distinct Rossmann-fold methyl transferases in LUCA.
Methylene transfer	Cyclopropane fatty acid synthase, EC:2.1.1.79	Rossmann-fold methyltransferase family	Derived from an ancient enzyme; not in LUCA
Aminoalkyl transfer ^1^	Nicotianamine synthase, EC:2.5.1.43	Rossmann-fold methyltransferase family with permuted order of sequence motifs	Derived from an ancient enzyme; not in LUCA
	ACC synthase, EC:4.4.1.14	PLP-dependent aminotransferase fold; the SAM-binding domain is derived from generic substrate-binding cleft	Derived from an ancient enzyme; not in LUCA
	Acyl-homoserine lactone synthase, EC:6.1.-	GNAT-type acetyltransferase fold; the SAM-binding domain is derived from generic substrate-binding cleft	Derived from an ancient enzyme; not in LUCA
Aminopropyl transfer	Spermidine synthase, EC:2.5.1.16	Rossmann-fold methyltransferase family, but the substrate is decarboxy-SAM	Probably in LUCA
Ribosyl transfer	tRNA-ribosyl transferase-isomerase, EC:5.-	QueA family; smaller β-barrel N-terminal domain and a larger C-terminal domain with α/β fold, distantly related to a TIM-barrel	Bacterial invention; not in LUCA
5'deoxyadenosyl transfer	5'-fluoro-5'-deoxy-adenosine synthase, EC:2.5.1.63	Two-domains; larger N-terminal domain has distant similarity to Rossmann-fold methyltransferases, smaller C-terminal domain is a β-barrel	Bacterial invention; not in LUCA
5'deoxyadenosyl radical synthesis	SAM radical enzymes	TIM-like α/β barrel with additional inserted elements. May have distant sequence similarity to TIM barrel of corrinoid methyltransferase (see text)	Probably in LUCA
SAM decarboxylation	SAM decarboxylase, EC:4.1.1.50	α/β/β/α sandwich in eukaryotes, apparently produced by duplication of a half-unit; stand-alone half-units exist in many bacteria and archaea	Probably in LUCA
De novo SAM synthesis	Methionine adenosyl transferase, EC:2.5.1.6	Unique fold: repeat of 3 β-α-β-β-α-β units	Probably in LUCA
Regulatory binding of SAM	Methionine repressor	All-α SAM-binding domain is derived from generic small molecule-binding domain	Bacterial innovation
	CBS domain	Mostly-β SAM-binding domain is derived from generic small molecule-binding domain	Not in LUCA
	Transcription factor mtTFB	Rossmann-fold methyltransferase family member that has lost catalytic activity	

1 An additional reaction in this class is synthesis of acp^3^U, a modified base in some tRNAs and rRNAs. This amino alkyl transfer requires SAM, but responsible protein has not been identified.

**Table 2:** Repertoire of SAM-binding proteins in the Last Universal Common Ancestor

**COG**	**Phyletic pattern parsimony**^1^	**Tree topology**^2^	**Protein product name and function**	**Fold class**
			**SAM-binding proteins in LUCA – best-supported COGs**	
**COG0030**	1/0	+	KsgA, Dimethyladenosine transferase (rRNA methylation)	Rossmann fold
**COG0144**	1/0	+	Sun, tRNA and rRNA cytosine-C5-methylases	Rossmann fold
**COG0192**	0/1 ^3^	+ ^3^	MetK, *S*-adenosylmethionine synthetase (releases SAM as product)	SAM synthase
**COG0421**	1/0	+	SpeE, Spermidine synthase	Rossmann fold
**COG0500**	1/0	+	SmtA, SAM-dependent methyltransferases	Rossmann fold
**COG0621**	1/0	+	MiaB, 2-methylthioadenine synthetase	Rossmann fold
**COG1180**	0/1	+	PflA, Pyruvate-formate lyase-activating enzyme	SAM radical (TIM barrel)
**COG1586**	0/1 ^3^	+ ^3^	SpeD, *S*-adenosylmethionine decarboxylase	SAM decarboxylase
**COG2890**	1/0	+	PrmC (HemK), Methylase of polypeptide chain release factors	Rossmann fold
			**SAM-binding proteins in LUCA – tentatively supported COGs**	
**COG0007**	1/0	?	CysG, Uroporphyrinogen-III methylase	SAM radical (TIM barrel)
**COG0220**	1/0	?	Predicted *S*-adenosylmethionine-dependent methyltransferase	Rossmann fold
**COG0293**	0/1	?	FtsJ, 23S rRNA methylase	Rossmann fold
**COG0320**	0/1	?	LipA, Lipoate synthase	SAM radical (TIM barrel)
**COG0502**	0/1	?	BioB, Biotin synthase and related enzymes	SAM radical (TIM barrel)
**COG0565**	0/1	?	LasT, rRNA methylases	SPOUT
**COG0685**	1/0	?	MetF, 5,10-methylenetetrahydrofolate reductase (binds SAM as inhibitor)	TIM barrel
**COG1189**	0/1	?	Predicted rRNA methylase	Rossmann fold
**COG2226**	1/0	?	UbiE, Methylase involved in ubiquinone/menaquinone biosynthesis	Rossmann fold

^1 ^Placement of COGs in LUCA based on weighted parsimony analysis of phyletic patterns [138]. 1/0 indicates that this gene has been tracked back to LUCA genome under all tested conditions; 0/1 indicates tentative placement of gene in LUCA under some but not all conditions.

^2 ^Phylogenetic analysis of each COG. Plus sign indicates tree topology that is fully consistent with gene presence in LUCA, and the question mark indicates a tentative support for the LUCA hypothesis (see Materials and Methods)

^3 ^The common ancestry and phylogeny of archaeal, bacterial, and eukaryotic homologs was established in this work (see text for details).

### SAM-binding modules derived from generic ligand-binding domains

#### ACC synthase

ACC synthase (*S*-adenosyl-L-methionine methylthioadenosine lyase, EC: 4.4.1.14, KOG0256) catalyses the rate-limiting step in biosynthesis of plant hormone ethylene by the α,γ-elimination of methylthioadenosine from SAM to produce 1-aminocyclopropane-1-carboxylate (ACC) [76]. ACC synthases require pyridoxal phosphate (PLP) for activity, and are related in sequence and structure to a large, diverse group of PLP-dependent transferases. The shared catalytic domain of this fold is of α/β/α type, with mixed central β-sheet of 7 strands (order 3245671), where strand 7 is antiparallel to the rest (SCOP fold: 53382; Figure 1j). Several residues are essential for the substrate binding (reviewed by Jakubowicz; numbered as in PDB structure 1B8G): Glu47 is responsible for putative ionic interaction with SAM; Ala46 and Arg407 interact with carboxypropyl moiety of SAM; Arg150 interacts with ribose moiety; and Ser18, Tyr19, Phe20, and Pro146 form hydrophobic pocket for the adenine ring of SAM [77]. Invariant Tyr85 is involved in the substrate recognition, and interacts with active-site Lys273 from the adjacent subunit (Lys273 forms a covalent Schiff base with PLP cofactor) [78,79].

Structure of ACC synthase is similar to other PLP-dependent transferases, such as transaminating aminotransferases, β-eliminating lyases, and cystathionine synthase. The only other enzyme in this group that binds SAM is 7,8-Diaminopelargonic acid (DAPA) synthase, which utilizes SAM in a different way than ACC synthase, namely as amino group donor in aminotransferase reaction. Both ACC synthase and DAPA synthase have similar active site residues involved in PLP cofactor binding. The interactions between SAM and DAPA synthase have not been studied in detail.

It is likely that ACC synthase and DAPA synthase evolved from other aminotransferases with different, perhaps broad, specificity, by accumulating changes in the ligand-binding region that increased its specificity towards SAM [80]. The evolutionary heritage of ACC synthase is manifest in the retained ability of the enzyme to catalyze slow transamination of substrates such as alanine [81]. Structural similarity between ACC-, DAPA-synthase and some other PLP-dependent enzymes (i.e. cystine C-S lyase – PDB structure 1ELQ; cystathionine β-lyase – PDB structure 1CL2) indicate that SAM binding in this case may have originated from ancestor with PLP-dependent binding of amino group of various sulfur containing amino acids or amino acid derivatives.

#### AHL synthase

Synthesis and detection of acyl-homoserine lactones (AHLs) enables many gram-negative bacteria to engage in quorum/diffusion sensing, an intercellular signaling mechanism that activates differentiation towards virulence and biofilm formation [82-84].

The AHL synthases (COG3916) catalyze acylation and lactonization of SAM, where the acyl group is provided by acylated acyl carrier protein (acyl-ACP) [85,86]. AHL synthases (SCOP family: 75508; Figure 1p) has acyl-CoA N-acyltransferase ("GNAT-like") fold: α/β/α sandwich with highly twisted β-sheet (SCOP fold: 55728). The conserved N-terminal residues: Arg23, Phe27, and Trp33 (numbered as in PDB structure 1RO5) form putative SAM binding pocket and undergo a dramatic conformational rearrangement upon acyl-ACP binding. This conformational change brings conserved residues of the putative SAM binding site in close proximity to the catalytic site [87]. Position of conserved β-bulge formed by Ser103 and Arg104 in β4 (numbered as in PDB structure 1RO5) distinguishes SAM-binding from other proteins with acyl-CoA-N-acyltransferase fold [86,87]. There is no detectable sequence or structural similarity between AHL synthases and other known SAM binding proteins, indicating independent origin of SAM binding in this fold. As with ACC synthase and DAPA synthase, the most likely mechanism of adaptation was by selecting relatively small changes in a generic ligand-binding region that increased relative affinity to SAM.

#### Met repressor

The *E. coli *MetJ repressor (Figure 1m; SCOP family: 100972; COG3060) uses SAM as a co-repressor to regulate the production of methionine. MetJ is a homodimeric, DNA-binding protein with ribbon-helix-helix fold. Co-repressor (SAM) binds to each monomer of the protein dimer at sites that lie on the opposite side of the protein from the DNA-binding motif. Binding of co-repressor affects DNA affinity, but apparently not specificity of MetJ [88-91]. Affinity of MetJ DNA binding is affected primarily by the positive charge associated with the ternary sulfur atom in co-repressor (SAM), which creates a region of positive electrostatic potential on the DNA binding surface overlapping the adjacent phosphodiester backbone in the region of the operator [92-94]. The SAM's adenine ring inserts itself deeply inside a hydrophobic pocket, consisted of side chains from both monomers. The positively charged sulfur of the SAM is greatly attracted by the net negatively charged C-terminal end of the β-helix, hence docking the SAM molecule in place. Electrostatic properties of SAM and its ability to serve as a regulatory feedback molecule in the common metabolic pathway of methionine synthesis probably played an important role in the emergence of this unique mode of SAM binding by MetJ.

MetJ is the only known SAM-binding representative of evolutionarily ancient ribbon-helix-helix (RHH) class of DNA-binding proteins [92,95]. Evolution of SAM cofactor binding in this protein was feasible because SAM adenosyl moiety fit into the cleft formed by both monomers, and its sulfonium center conformation was able to adapt to non-catalytic electrostatic interactions with MetJ repressor.

#### CBS

CBS-domains (COG0517) are widely distributed in all divisions of Life, in the form of fusions with various unrelated proteins, where they usually form tandem pairs. Binding of the adenosyl-containing molecules, such as ATP, AMP, and SAM by CBS-domains is important for their function as energy or redox status-sensing modules [96-98]. Some CBS-domains also binds single stranded nucleic acids [99].

In general, a tandem of CBS domains (encoded by ~120 aa) folds into one domain with a β-sandwich and 4 α-helices extending from one edge (Figure 1o). CBS domains within each pair are asymmetric. CBS-domain is common in multidomain proteins (i.e.: 15 in Bacteria and 9 in Archaea [100]) and is probably derived from generic small molecule-binding domain. The mode of SAM binding to CBS domains remains unknown.

### Rare folds

#### Porphyrin C-methyltransferase

Porphyrin C-methyltransferases are a family of proteins involved in biosynthesis of tetrapyrroles, which are used in chelation of metal ion. Representative structures of Cbif and CysG with bound reaction product (SAH) are known. They have the same fold, which consists of two dissimilar α/β domains. Domain 1 has parallel sheet of 5 strands (order 32415) sandwiched between 3 α-helices; domain 2 has mixed sheet of 5 strands (also sandwiched between 3 α-helices), order 12534; strands 4 & 5 are antiparallel to the rest; (Figure 1d) [101,102]. The reaction product is bound in a large pocket between the N and C-terminal domains. Binding pocket contains conserved glycine-rich loop GAGPGD, similarly to Rossmann-fold methyltransferases, but in crystal structures of porphyrin C-methyltransferases (i.e. PDB structure 1CBF, 1PJQ, and 1S4D) glycine-rich loop does not contact methionine moiety of SAH, at least in the absence of the precorrin substrate. Instead, Pro30 from the glycine-rich loop (numbered as in PDB structure 1CBF) forms hydrogen bond with adenosyl moiety. Other conserved residues participate in (SAH) binding: adenosyl moiety hydrogen-bond with conserved Ala213, ribosyl moiety hydrogen bound with conserved hydrophobic Leu184 and Ala241; carboxyl group bind Asp103 from conserved Gly-Asp-Pro motif and also Tyr131; the amino group hydrogen-bond with conserved hydrophobic Met106. Near the entrance of SAM binding cleft there is conserved aromatic residue (Tyr107) positioned next to the sulfur of SAH.

Despite structural similarity to Rossmann-fold methyltransferases and local sequence similarity in the glycine-rich loop, porphyrin C-methyltransferases have distinct mode of SAM binding. The evolutionary relationships between porphyrin methyltransferases and other SAM-binding (or indeed any other) proteins remain unclear.

#### Met synthase activation domain

B_12_-dependent methionine synthases are large multidomain proteins, which catalyze a multistep reaction of the transfer of a methyl group from 5-methyltetrahydrofolate to homocysteine. The C-terminal domain of Met synthase is involved in reactivation of spontaneously oxidized coenzyme B_12_, and therefore is required for the catalysis. This activation domain (pfam02965 and C-terminal part of COG1410) has an unusual fold: β-α(2)-β(2)-α(2)-β-α-β; antiparallel β-sheet; order 12354 (Figure 1e; SCOP fold: 56506). Large conformational changes are required for reactivation reaction to occur [103-105].

This domain binds SAM in a shallow groove along the edges of the β-strands. There are several conserved residues involved in SAM binding (numbered as in PDB structure 1MSK): Asp946 and Glu1101 bind carboxypropyl moiety; Arg1134 and Tyr1130 (later via water mediated interactions) bind ribosyl moiety; Tyr1139 and Tyr1189 participate in stacking interactions with adenosyl moiety; Tyr1190 main chain (or Phe in other known sequences) hydrogen bond with adenosyl moiety of SAM. This unusual domain is found in bacterial and metazoan proteins. Interestingly, Met synthases of thermophilic bacteria lack the SAM-binding/activation domain altogether. Presumably, the lack of the transferase domain activity is compensated for by the methyltransferase protein also involved in the same reaction [106]. This particular adaptation in thermophiles seems to be partly due to requirement for greater thermal stability of the enzyme – making large conformational changes required for reactivation less favorable.

#### Divergence and convergence in evolution of SAM binding

The known SAM-binding proteins belong to 15 distinct superfamilies/folds, which comprise at least 20 more sequence families (Figure 1 and Table 4), that is: **1 **– Rossmann fold (methyltransferase, methylene transferase, nicotianamine synthase, spermidine synthase, spermine synthase, and acalcynomycin-10-hydroxylase), **2 **– Met synthase activating domain, **3 **– porphyrin C-methyltransferase, **4 **– SPOUT methyltransferase, **5 **– SET domain methyltransferase, **6 **– isoprenylcysteine carboxyl methyltransferase, **7 **– fluorinating enzyme, **8 **– tRNA ribosyltransferase-isomerase (QueA), **9 **– SAM decarboxylase, **10 **– SAM synthetase, **11 **– ACC synthase and DAPA synthase, **12 **– N-acyl-homoserine lactone synthase, **13 **– Met repressor, **14 **– CBS domain, **15 **– SAM-dependent radical.

These folds belong to all four large structural classes [107], though there is a distinct excess of α-β and especially α/β folds.

Comparison of fold classes and molecular functions reveals a broad picture of complex interplay between sequence divergence and functional convergence in the evolution of SAM-binding proteins and SAM-dependent molecular functions. On the whole, the assemblage of SAM-binding proteins is extremely heterogeneous. There are huge, apparently monophyletic, superfamilies, which in turn belong to even larger superfolds (Class I methyltransferases from a Rossmann-like superfold is an example), and there are small families with unique folds. There are molecular functions confined to just one superfamily, as in the case of SAM radical formation, which thus far is known to be performed only by enzymes belonging to large and diverse, yet apparently monophyletic, TIM-barrel-like SAM-radical fold. On the other hand, there is SAM-dependent methylation, performed by at least five classes of enzymes, which represent two completely different fold classes and may have been "invented" three or four times (ref. [12] and this study).

Duplication of protein domains is relatively common in both prokaryotes and eukaryotes, for example at least 58% of the domains in Mycoplasma [108] and 98% of the domains in humans [109-111] are duplicates. Several SAM-binding proteins appear to have evolved by ancient domain duplications. Examples include SET-domain methyltransferase [71], SAM synthase, where triplication of a basic wedge-shaped module is likely, and eukaryal SAM decarboxylase (this study). Here again, there is no strict correlation between that mechanism of protein emergence and its further evolution trajectory: SAM synthase and SAM decarboxylase have most likely persisted in evolution as single- or low-copy, vertically transmitted genes since the Last Universal Common Ancestor, whereas SET methyltransferases appear to be an eukaryotic innovation that experienced lineage-specific expansions, significant diversification of substrate specificity, and occasional horizontal transfer to prokaryotes.

Examination of the largest, most diverse set of SAM-dependent enzymes unified by common biochemical function, namely, SAM-dependent methyltransferases, shows more of the same trend. All methyltransferases belong to the same EC class (EC: 2.1.1.-), but they comprise five structural families (reviewed in ref. [12]), which appear to lack clearly discernible common ancestor (with the possible exception of Class I and SPOUT methyltransferase, see above). Three classes, I, IV, and V, are large, found across broad groups of genomes, and, at least in the cases of classes I and V, in multiple genomic contexts, including various protein fusions. In contrast, class II is restricted to just one specialized enzymatic system, methionine synthase, with very specific domain composition. Class I displays a huge variety of substrate specificities, whereas the substrates of other classes are much more narrowly defined (tetrapyrroles for class III, mostly rRNAs and tRNAs for class IV, and short list of proteins for class V). Interestingly, each of these classes of substrates are also targeted by class I enzymes. For example, although most of precorrin methyltransferases are class III enzymes, precorrin-C6 methyltransferase CbiT is a typical Class-I Rossmannoid [112]. Similarly, though most histone lysine N-methyltransferases are class V (SET-domain proteins), the Dot1 histone H3-Lys79 N-methyltransferase belongs to Class I [113]. In at least one case, exactly the same base in tRNA is methylated by Class I methyltransferase in bacteria and by class IV enzyme in archaea [114], suggesting either parallel evolution of different molecular solutions for the same task, or functional takeover by an unrelated gene.

### Phyletic distribution and phylogeny of SAM-binding proteins suggests multiple roles for SAM in the Last Universal Common Ancestor

SAM-binding proteins perform an unprecedented variety of chemical reactions, and belong to about 22 distinct sequence and structural families of proteins. It has been noted that some of these groups of proteins (most notably, Rossmann-fold methyltransferases) are extremely ancient and were more than likely represented by multiple paralogs in the life forms predating the divergence of bacteria, archaea, and eukarya [5]. On the other hand, some of the SAM-binding proteins appear to have been invented later in evolution. Such scenarios have been documented, for example, for SET-domain methyltransferase family, which is thought to have emerged in early eukaryotes and then passed to a few bacteria by lateral gene transfer, while greatly expanding in size in multicellular eukaryotes [71], and are also likely for Met repressor, which is a member of the large ligand-binding family essentially confined to bacteria [115]. A complex picture of gains, losses, and lineage-specific expansions of genes coding for SAM-binding proteins is illustrated in Table 3.

**Table 3:** Counts of each family in selected genomes

**Protein family name and number of genes (proteins) per genome**							**Additional information about counts of each family in selected genomes.**
**HUMAN**	**ARATH**	**YEAST**	**PSEAE**	**MYCTU**	**SULSO**	**HELPY**	
**1. **Rossmann fold MTase							
49 (64)	70 (81)	30	55	37	31	26	Rossmann fold methyltransferase (MTase) makes the list of 10 most commonly used sequence and structure families [15]. In analyzed genomes, 4.6% of Rossmann fold MTase does not have motif-I (Gly-rich) and may lack MTase activity. **Fold: **Rossmann-fold.
**2. **Met synthase activating domain							
1	0	0	1	1	0	0	Present in many bacteria and metazoan. **Fold: **Met synthase activation domain-like.
**3. **MTase class III							
1	3 (4)	2	8	6	7	1	Human genome encodes protein similar to diphthine synthase but not other Class-III MTases (i.e. from tetrapyrrole biosynthesis pathway) identified in analyzed genomes. **Fold: **Tetrapyrrole-methyltransferase.
**4. **SPOUT MTase							
3	5	2	4	6	1	2	Every analyzed here genome has at least one putative SPOUT rRNA MTase, in archaea the same base of tRNA is methylated by Rossmann-fold instead of SPOUT MTase. **Fold: **α/β knot.
**5. **SET domain MTase							
25 (34)	34 (41)	5	0	0	0	0	Present in all Eukaryota with sporadic lateral transfer to bacteria and archaea (but not identified in prokaryotic genomes analyzed here). In *S. cerevisiae *all selected sequences similar to SET-domain MTases are conserved within smart00317 domain (in contrast to SET-domain proteins other then MTases). **Fold: **β-clip.
**6. **methylene transferase							
0	4	0	1	10	0	1	This enzyme performs cyclopropane fatty acid synthesis and is important for *Mycobacterium *survival. **Fold: **Rossmann-fold.
**7. **Nicotianamine synthase							
0	4	0	0	0	0	0	This is plant enzyme has similarity to proteins with unknown function infrequently distributed in bacteria, archaea and fungi (discussed earlier). **Fold: **Rossmann-fold (predicted).
**8. **spermidine synthase &**9. **spermine synthase							
1	3	2	3	1	1	1	The mammalian enzyme is highly specific but the bacterial enzyme can use other acceptors then SAM and can synthesize spermine. Spermidine synthase but not spermine synthase is essential for survival of *Arabidopsis *and *S. cerevisiae *[142-144]. **Fold: **Rossmann-fold.
**10. **aclacynomycin-10-hydroxylase							
0	0	0	0	0	0	0	Was found in *Streptomyces purpurascens *only. **Fold: **Rossmann-fold.
**11. **isoprenylcysteine *O*-MTase							
2	2	1	0	3	0	0	ICMT enzymes are present in all eukaryotic organisms [145]. **Fold**: unknown (predicted: all-α).
**12. **fluorinating enzyme							
0	0	0	0	0	0	0	Gene encoding fluorinating enzyme was identified in *Streptomyces cattleya *but not yet in plants synthesizing fluorinated metabolites. **Fold: **Rossmann-like and β-barrel.
**13. **QueA							
0	0	0	1	0	0	1	QueA (tRNA ribosyltransferase – isomerase) homologs are found only in bacteria. **Fold: **QueA-like (TIM-barrel and β-barrel).
**14. **SAM decarboxylase							
1	4	1	2	?	2	?	SAM decarboxylase activity has been purified from all three domains of Life. However *M. tuberculosis *and *H. pylori *lack clearly identifiable homologue of this enzyme. **Fold: **SAM-decarboxylase.
**15. **SAM synthetase							
2	4 (5)	2	1	1	1	1	SAM synthases from bacteria and eukaryotes are closely related at the sequence level and have very similar structures [73]. SAM synthase is involved in development and abiotic stress tolerance in plants and have complex expression pattern [146, 147]. **Fold: **SAM-synthetase.
**16. **ACC synthase							
2	12	0	0	0	0	0	ACC is the precursor of important plant hormone – ethylene. Human homologs have different function. **Fold: **PLP-dependent transferases.
**17. **N-acyl-homoserine lactone synthase							
0	0	0	2	0	0	0	Found in some bacteria only. **Fold: **GNAT.
**18. **Met repressor							
0	0	0	0	0	0	0	Found in some enterobacteria and gamma proteobacteria only. **Fold: **ribbon-helix-helix.
**19. **CBS domain							
5	10	5	7	4	9	3	Numbers of SAM-binding CBS domains presented here are approximate because of strong similarity to CBS domains binding other adenosine derivatives. **Fold: **CBS-domain.
**20. **DAPA synthase							
0	1	1	6	2	0	1	Biotin biosynthesis is unique to plants, some fungi and most bacteria. **Fold: **PLP-dependent transferases.
**21. **SAM-dependent radical							
9 (13)	13 (14)	5	18	11	24	11	Human lacks SAM-dependent radical enzyme from biotin and thiamine biosynthetic pathways. Those enzymes generate highly oxidizing 5'-deoxyadenosyl radical in an anaerobic reducing environment, and utilize this radical as catalytic and stoichiometric oxidant in many different enzymatic reactions [148]. Those enzymes are essential for anaerobic growth. **Fold:**TIM-barrel.

Number of genes and proteins encoded by selected genomes were counted for each SAM-binding protein family. **SULSO **– *Sulfolobus solfataricus *(Archaea), **PSEAE **– *Pseudomonas aeruginosa *(Gamma proteobacteria), **HELPY **– *Helicobacter pylori *J99 (Proteobacteria), **MYCTU **– *Mycobacterium tuberculosis *(Actinobacteria), **YEAST **– *Saccharomyces cerevisiae *(Eukaryota), **ARATH **– *Arabidopsis thaliana *(Eukaryota), **HUMAN **– *Homo sapiens *(Eukaryota).

We performed a more detailed examination of evolutionary trajectories of SAM-binding proteins, using information from the NCBI COG database. Each COG is a set of orthologous genes in completely sequenced genomes, along with lineage-specific paralogs. A COG is characterized by phyletic pattern, which is the set of genomes that has at least one member of this COG, and by sequence-based phylogenetic tree of COG members [116]. This information can be used, in conjunction with the consensus phylogenetic tree of the completely sequence genomes, to infer the presence or absence of a COG in an ancestral life form. We used a relatively conservative estimate, allowing for occasional horizontal gene transfer and demanding a complete agreement between species' tree, gene family tree, and phyletic pattern (see Methods for more detail). Under these conditions, the SAM-binding complement of LUCA proteins consists of 9 ancestral COGs (Table 2).

Although 6 of 9 COGs in LUCA represent proteins with Rossmann-like fold, the remaining three folds are all different. It should be noted that the set of 9 COGs is almost certainly an underestimation of the SAM-binding proteome in LUCA. Evolutionary model allowing more frequent horizontal gene transfer and/or non-orthologous gene displacements, as well as slight disagreement between different lines of evidence, will increase the list of COGs that can be placed in LUCA genome. The specificity of these additional enzymes varies, although the modification of translation apparatus continues to figure prominently in the increased set; the diversity of folds, however, appears to increase only slightly, mostly due to addition of the SPOUT fold.

An already-diverse group of Rossmann-like SAM-dependent transferases, a variation of TIM-barrel, and two unique αββα architectures thus appear to represent the best-supported ancestral set of SAM-binding proteins. Although less diverse than the present-day variety of SAM-binding proteins, this set is far from simple. Interestingly, it consists of proteins with α/β architecture and is depleted of all-α and all-β proteins, as seems to be the case for other categories of ancestral enzymes [117] and perhaps non-enzymatic proteins too [118].

The substrates and molecular functions of many of these enzymes are hard to ascertain. Ancient enzymes may have had broader specificities than their present-day descendants [119], but several pathways nevertheless emerge from the analysis of the SAM-binding proteome of the LUCA (Table 2). The common ancestor of bacteria, archaea and eukarya appears to have been able to synthesize SAM de novo, from ATP and methionine; to use it for methylation of RNA bases and, probably, proteins such as translation factors; to decarboxylate SAM; and to synthesize polyamines with the aid of dcSAM. In addition, LUCA had the capacity for generating SAM radicals.

Slightly less restrictive evolutionary model enlarges the set of SAM-binding proteins in LUCA, mostly by increasing the number of paralogs in Class I MT and SAM-radical families, but also by supplementing the set with SPOUT methyltransferases. Further sequence and structure comparison may provide for more detailed understanding of these ancestors, perhaps even to the point of reconstructing the ancestral sequences and studying the ancient SAM-binding proteins in the laboratory.

## Conclusion

There are 15 distinct superfamilies of SAM-binding proteins, at least 5 of which may have been represented in the last common ancestor.

## Methods

Analysis of multiple sequences in the batch mode was handled using the SEALS package [120].

Iterative database searches with position-specific weight matrices (PSSMs) were performed using the PSI-BLAST program, with the expectation value for inclusion into the PSSM (-h parameter) set at 0.01, unless otherwise indicated [121]. Additional profile searches were carried out using hidden Markov models generated from alignments of protein domains using the hmmsearch program of the HMMER2 package [122].

Multiple alignments of protein sequences were constructed in an iterative fashion, alternating between sequence and structure alignments [123]. Structural alignments of representative structures from the SCOP families [107] were produced using CE-MC [124] and DALI [125]. The muscle program [126] was used to refine all alignments.

The 3-dimensional structures of proteins were manipulated using the Rasmol program and ribbon diagrams were drawn using the PyMOL program [127].

The Metaserver approach [56] was used to interrogate the network of programs that perform secondary structure prediction and tertiary fold-recognition of proteins by a variety of probabilistic matching and energy calculations (here we used predictions from: PSIPRED [128], PROFsec [129], SAM-T02 [130], FFAS03 [131], 3DPSSM [132], BIOINBGU [133], ORFeus [134], FUGUE [135], and Pcons server [136]).

Topology diagrams were created by using TOPS [137].

The inference of the ancestral presences/absences of the COGs has been done by Mirkin et al. [138]; their model was used in this study, with modifications described in Mushegian [118].

## List of abbreviations used

SAM: *S*-adenosylmethionine

PDB: Protein Data Bank

SCOP: Structural Classification of Proteins

## Authors' contributions

ARM: initiated and supervised the study, wrote parts of the manuscript and performed the analysis of phyletic distribution and phylogeny. PZK: participated in the design of the study, wrote parts of the manuscript and performed the large scale sequence and structure comparison. Both authors contributed to the writing of the manuscript, interpreted all data, and approved the final version.

## References

[B1] Waddell TG, Eilders LL, Patel BP, Sims M (2000). Prebiotic methylation and the evolution of methyl transfer reactions in living cells.. Orig Life Evol Biosph.

[B2] Thomas DJ, Waters SB, Styblo M (2004). Elucidating the pathway for arsenic methylation.. Toxicol Appl Pharmacol.

[B3] Wuosmaa AM, Hager LP (1990). Methyl chloride transferase: a carbocation route for biosynthesis of halometabolites.. Science.

[B4] Saxena D, Aouad S, Attieh J, Saini HS (1998). Biochemical characterization of chloromethane emission from the wood-rotting fungus Phellinus pomaceus. Appl Environ Microbiol.

[B5] Anantharaman V, Koonin EV, Aravind L (2002). Comparative genomics and evolution of proteins involved in RNA metabolism.. Nucleic Acids Res.

[B6] Hopper AK, Phizicky EM (2003). tRNA transfers to the limelight.. Genes Dev.

[B7] Kouzarides T (2002). Histone methylation in transcriptional control.. Curr Opin Genet Dev.

[B8] Aravind L, Koonin EV (1999). Novel predicted RNA-binding domains associated with the translation machinery.. J Mol Evol.

[B9] Romano JD, Michaelis S (2001). Topological and mutational analysis of Saccharomyces cerevisiae Ste14p, founding member of the isoprenylcysteine carboxyl methyltransferase family. Mol Biol Cell.

[B10] Anderson JL, Frase H, Michaelis S, Hrycyna CA (2005). Purification, functional reconstitution, and characterization of the Saccharomyces cerevisiae isoprenylcysteine carboxylmethyltransferase Ste14p. J Biol Chem.

[B11] Peterson YK, Winter-Vann AM, Casey PJ (2005). Icmt. AfCS-Nature Molecule Pages.

[B12] Schubert HL, Blumenthal RM, Cheng X (2003). Many paths to methyltransfer: a chronicle of convergence.. Trends Biochem Sci.

[B13] Fontecave M, Atta M, Mulliez E (2004). S-adenosylmethionine: nothing goes to waste.. Trends Biochem Sci.

[B14] Lesk AM (1995). NAD-binding domains of dehydrogenases.. Curr Opin Struct Biol.

[B15] Wolf YI, Brenner SE, Bash PA, Koonin EV (1999). Distribution of protein folds in the three superkingdoms of life.. Genome Res.

[B16] Martin JL, McMillan FM (2002). SAM (dependent) I AM: the S-adenosylmethionine-dependent methyltransferase fold.. Curr Opin Struct Biol.

[B17] Bujnicki JM (1999). Comparison of protein structures reveals monophyletic origin of the AdoMet-dependent methyltransferase family and mechanistic convergence rather than recent differentiation of N4-cytosine and N6-adenine DNA methylation.. In Silico Biol.

[B18] Posfai J, Bhagwat AS, Posfai G, Roberts RJ (1989). Predictive motifs derived from cytosine methyltransferases.. Nucleic Acids Res.

[B19] Fauman EB, Blumenthal RM, Cheng X, Cheng X and Blumenthal RM (1999). Structure and evolution of AdoMet-dependent MTases.. In S-Adenosylmethionine-dependent methyltransferases: structures and functions.

[B20] Bujnicki JM (2002). Sequence permutations in the molecular evolution of DNA methyltransferases.. BMC Evol Biol.

[B21] Sankpal UT, Rao DN (2002). Mutational analysis of conserved residues in HhaI DNA methyltransferase. Nucleic Acids Res.

[B22] Cheng X (1995). Structure and function of DNA methyltransferases.. Annu Rev Biophys Biomol Struct.

[B23] Kumar S, Cheng X, Klimasauskas S, Mi S, Posfai J, Roberts RJ, Wilson GG (1994). The DNA (cytosine-5) methyltransferases. Nucleic Acids Res.

[B24] Bugl H, Fauman EB, Staker BL, Zheng F, Kushner SR, Saper MA, Bardwell JC, Jakob U (2000). RNA methylation under heat shock control.. Mol Cell.

[B25] Feder M, Pas J, Wyrwicz LS, Bujnicki JM (2003). Molecular phylogenetics of the RrmJ/fibrillarin superfamily of ribose 2'-O-methyltransferases. Gene.

[B26] Liu J, Mushegian A (2003). Three monophyletic superfamilies account for the majority of the known glycosyltransferases.. Protein Sci.

[B27] Cheek S, Zhang H, Grishin NV (2002). Sequence and structure classification of kinases.. J Mol Biol.

[B28] Unligil UM, Zhou S, Yuwaraj S, Sarkar M, Schachter H, Rini JM (2000). X-ray crystal structure of rabbit N-acetylglucosaminyltransferase I: catalytic mechanism and a new protein superfamily.. EMBO J.

[B29] Qasba PK, Ramakrishnan B, Boeggeman E (2005). Substrate-induced conformational changes in glycosyltransferases. Trends Biochem Sci.

[B30] Nes WD, Marshall JA, Jia Z, Jaradat TT, Song Z, Jayasimha P (2002). Active site mapping and substrate channeling in the sterol methyltransferase pathway.. J Biol Chem.

[B31] Zubieta C, He XZ, Dixon RA, Noel JP (2001). Structures of two natural product methyltransferases reveal the basis for substrate specificity in plant O-methyltransferases.. Nat Struct Biol.

[B32] Huang CC, Smith CV, Glickman MS, Jacobs JWR, Sacchettini JC (2002). Crystal structures of mycolic acid cyclopropane synthases from Mycobacterium tuberculosis.. J Biol Chem.

[B33] Takahashi M, Terada Y, Nakai I, Nakanishi H, Yoshimura E, Mori S, Nishizawa NK (2003). Role of nicotianamine in the intracellular delivery of metals and plant reproductive development.. Plant Cell.

[B34] Dela Vega AL, Delcour AH (1996). Polyamines decrease Escherichia coli outer membrane permeability. J Bacteriol.

[B35] Nikaido H (2003). Molecular basis of bacterial outer membrane permeability revisited. Microbiol Mol Biol Rev.

[B36] Korolev S, Ikeguchi Y, Skarina T, Beasley S, Arrowsmith C, Edwards A, Joachimiak A, Pegg AE, Savchenko A (2002). The crystal structure of spermidine synthase with a multisubstrate adduct inhibitor.. Nat Struct Biol.

[B37] Jansson A, Koskiniemi H, Erola A, Wang J, Mantsala P, Schneider G, Niemi J (2005). Aclacinomycin 10-hydroxylase is a novel substrate-assisted hydroxylase requiring S-adenosyl-L-methionine as cofactor. J Biol Chem.

[B38] McCulloch V, Shadel GS (2003). Human mitochondrial transcription factor B1 interacts with the C-terminal activation region of h-mtTFA and stimulates transcription independently of its RNA methyltransferase activity.. Mol Cell Biol.

[B39] Bujnicki JM (2001). In silico analysis of the tRNA:m1A58 methyltransferase family: homology-based fold prediction and identification of new members from Eubacteria and Archaea. FEBS Lett.

[B40] Anderson J, Phan L, Hinnebusch AG (2000). The Gcd10p/Gcd14p complex is the essential two-subunit tRNA(1-methyladenosine) methyltransferase of Saccharomyces cerevisiae. Proc Natl Acad Sci U S A.

[B41] Bujnicki JM, Feder M, Radlinska M, Blumenthal RM (2002). Structure prediction and phylogenetic analysis of a functionally diverse family of proteins homologous to the MT-A70 subunit of the human mRNA:m(6)A methyltransferase. J Mol Evol.

[B42] Bujnicki JM, Rychlewski L (2002). RNA:(guanine-N2) methyltransferases RsmC/RsmD and their homologs revisited-bioinformatic analysis and prediction of the active site based on the uncharacterized Mj0882 protein structure.. BMC Bioinformatics.

[B43] Chen ZX, Mann JR, Hsieh CL, Riggs AD, Chedin F (2005). Physical and functional interactions between the human DNMT3L protein and members of the de novo methyltransferase family. J Cell Biochem.

[B44] Gowher H, Liebert K, Hermann A, Xu G, Jeltsch A (2005). Mechanism of stimulation of catalytic activity of Dnmt3A and Dnmt3B DNA-(cytosine-C5)-methyltransferases by Dnmt3L. J Biol Chem.

[B45] Bourc'his D, Bestor TH (2004). Meiotic catastrophe and retrotransposon reactivation in male germ cells lacking Dnmt3L. Nature.

[B46] Dong C, Huang F, Deng H, Schaffrath C, Spencer JB, O'Hagan D, Naismith JH (2004). Crystal structure and mechanism of a bacterial fluorinating enzyme.. Nature.

[B47] Anantharaman V, Koonin EV, Aravind L (2002). SPOUT: a class of methyltransferases that includes spoU and trmD RNA methylase superfamilies, and novel superfamilies of predicted prokaryotic RNA methylases.. J Mol Microbiol Biotechnol.

[B48] Jackman JE, Montange RK, Malik HS, Phizicky EM (2003). Identification of the yeast gene encoding the tRNA m1G methyltransferase responsible for modification at position 9. RNA.

[B49] Ginalski K, von Grotthuss M, Grishin NV, Rychlewski L (2004). Detecting distant homology with Meta-BASIC. Nucleic Acids Res.

[B50] Ahn HJ, Kim HW, Yoon HJ, Lee BI, Suh SW, Yang JK (2003). Crystal structure of tRNA(m1G37)methyltransferase: insights into tRNA recognition.. EMBO J.

[B51] Gerstein M (1997). A structural census of genomes: comparing bacterial, eukaryotic, and archaeal genomes in terms of protein structure. J Mol Biol.

[B52] Nagano N, Orengo CA, Thornton JM (2002). One fold with many functions: the evolutionary relationships between TIM barrel families based on their sequences, structures and functions.. J Mol Biol.

[B53] Sofia HJ, Chen G, Hetzler BG, Reyes-Spindola JF, Miller NE (2001). Radical SAM, a novel protein superfamily linking unresolved steps in familiar biosynthetic pathways with radical mechanisms: functional characterization using new analysis and information visualization methods.. Nucleic Acids Res.

[B54] Ollagnier-de Choudens S, Sanakis Y, Hewitson KS, Roach P, Munck E, Fontecave M (2002). Reductive cleavage of S-adenosylmethionine by biotin synthase from Escherichia coli. J Biol Chem.

[B55] Nicolet Y, Drennan CL (2004). AdoMet radical proteins-from structure to evolution-alignment of divergent protein sequences reveals strong secondary structure element conservation. Nucleic Acids Res.

[B56] Ginalski K, Elofsson A, Fischer D, Rychlewski L (2003). 3D-Jury: a simple approach to improve protein structure predictions. Bioinformatics.

[B57] Marton LJ, Pegg AE (1995). Polyamines as targets for therapeutic intervention. Annu Rev Pharmacol Toxicol.

[B58] Gerner EW, Meyskens FLJ (2004). Polyamines and cancer: old molecules, new understanding. Nat Rev Cancer.

[B59] Kim AD, Graham DE, Seeholzer SH, Markham GD (2000). S-Adenosylmethionine decarboxylase from the archaeon Methanococcus jannaschii: identification of a novel family of pyruvoyl enzymes. J Bacteriol.

[B60] Sekowska A, Coppee JY, Le Caer JP, Martin-Verstraete I, Danchin A (2000). S-adenosylmethionine decarboxylase of Bacillus subtilis is closely related to archaebacterial counterparts. Mol Microbiol.

[B61] Ekstrom JL, Mathews II, Stanley BA, Pegg AE, Ealick SE (1999). The crystal structure of human S-adenosylmethionine decarboxylase at 2.25 A resolution reveals a novel fold. Structure Fold Des.

[B62] Toms AV, Kinsland C, McCloskey DE, Pegg AE, Ealick SE (2004). Evolutionary links as revealed by the structure of Thermotoga maritima S-adenosylmethionine decarboxylase. J Biol Chem.

[B63] Lu ZJ, Markham GD (2004). Catalytic properties of the archaeal S-adenosylmethionine decarboxylase from Methanococcus jannaschii. J Biol Chem.

[B64] Bennett EM, Ekstrom JL, Pegg AE, Ealick SE (2002). Monomeric S-adenosylmethionine decarboxylase from plants provides an alternative to putrescine stimulation. Biochemistry.

[B65] Trievel RC, Beach BM, Dirk LM, Houtz RL, Hurley JH (2002). Structure and catalytic mechanism of a SET domain protein methyltransferase.. Cell.

[B66] Yokoyama A, Wang Z, Wysocka J, Sanyal M, Aufiero DJ, Kitabayashi I, Herr W, Cleary ML (2004). Leukemia proto-oncoprotein MLL forms a SET1-like histone methyltransferase complex with menin to regulate Hox gene expression. Mol Cell Biol.

[B67] Kouskouti A, Scheer E, Staub A, Tora L, Talianidis I (2004). Gene-specific modulation of TAF10 function by SET9-mediated methylation. Mol Cell.

[B68] Chuikov S, Kurash JK, Wilson JR, Xiao B, Justin N, Ivanov GS, McKinney K, Tempst P, Prives C, Gamblin SJ, Barlev NA, Reinberg D (2004). Regulation of p53 activity through lysine methylation.. Nature.

[B69] Xiao B, Jing C, Wilson JR, Walker PA, Vasisht N, Kelly G, Howell S, Taylor IA, Blackburn GM, Gamblin SJ (2003). Structure and catalytic mechanism of the human histone methyltransferase SET7/9.. Nature.

[B70] Manzur KL, Farooq A, Zeng L, Plotnikova O, Koch AW, Zhou MM, Sachchidanand (2003). A dimeric viral SET domain methyltransferase specific to Lys27 of histone H3.. Nat Struct Biol.

[B71] Aravind L, Iyer LM (2003). Provenance of SET-domain histone methyltransferases through duplication of a simple structural unit. Cell Cycle.

[B72] Iyer LM, Aravind L (2004). The emergence of catalytic and structural diversity within the beta-clip fold. Proteins.

[B73] Sanchez-Perez GF, Bautista JM, Pajares MA (2004). Methionine adenosyltransferase as a useful molecular systematics tool revealed by phylogenetic and structural analyses. J Mol Biol.

[B74] Komoto J, Yamada T, Takata Y, Markham GD, Takusagawa F (2004). Crystal structure of the S-adenosylmethionine synthetase ternary complex: a novel catalytic mechanism of S-adenosylmethionine synthesis from ATP and Met.. Biochemistry.

[B75] Graham DE, Bock CL, Schalk-Hihi C, Lu ZJ, Markham GD (2000). Identification of a highly diverged class of S-adenosylmethionine synthetases in the archaea. J Biol Chem.

[B76] Huai Q, Xia Y, Chen Y, Callahan B, Li N, Ke H (2001). Crystal structures of 1-aminocyclopropane-1-carboxylate (ACC) synthase in complex with aminoethoxyvinylglycine and pyridoxal-5'-phosphate provide new insight into catalytic mechanisms. J Biol Chem.

[B77] Jakubowicz M (2002). Structure, catalytic activity and evolutionary relationships of 1-aminocyclopropane-1-carboxylate synthase, the key enzyme of ethylene synthesis in higher plants. Acta Biochim Pol.

[B78] Capitani G, Eliot AC, Gut H, Khomutov RM, Kirsch JF, Grutter MG (2003). Structure of 1-aminocyclopropane-1-carboxylate synthase in complex with an amino-oxy analogue of the substrate: implications for substrate binding. Biochim Biophys Acta.

[B79] Tsuchisaka A, Theologis A (2004). Heterodimeric interactions among the 1-amino-cyclopropane-1-carboxylate synthase polypeptides encoded by the Arabidopsis gene family. Proc Natl Acad Sci U S A.

[B80] Eliot AC, Kirsch JF (2003). Avoiding the road less traveled: how the topology of enzyme-substrate complexes can dictate product selection. Acc Chem Res.

[B81] Feng L, Geck MK, Eliot AC, Kirsch JF (2000). Aminotransferase activity and bioinformatic analysis of 1-aminocyclopropane-1-carboxylate synthase. Biochemistry.

[B82] Fuqua C, Parsek MR, Greenberg EP (2001). Regulation of gene expression by cell-to-cell communication: acyl-homoserine lactone quorum sensing. Annu Rev Genet.

[B83] Redfield RJ (2002). Is quorum sensing a side effect of diffusion sensing?. Trends Microbiol.

[B84] Lerat E, Moran NA (2004). The evolutionary history of quorum-sensing systems in bacteria. Mol Biol Evol.

[B85] Val DL, Cronan JEJ (1998). In vivo evidence that S-adenosylmethionine and fatty acid synthesis intermediates are the substrates for the LuxI family of autoinducer synthases. J Bacteriol.

[B86] Watson WT, Minogue TD, Val DL, von Bodman SB, Churchill ME (2002). Structural basis and specificity of acyl-homoserine lactone signal production in bacterial quorum sensing. Mol Cell.

[B87] Gould TA, Schweizer HP, Churchill ME (2004). Structure of the Pseudomonas aeruginosa acyl-homoserinelactone synthase LasI. Mol Microbiol.

[B88] He YY, Stockley PG, Gold L (1996). In vitro evolution of the DNA binding sites of Escherichia coli methionine repressor, MetJ. J Mol Biol.

[B89] Saint-Girons I, Belfaiza J, Guillou Y, Perrin D, Guiso N, Barzu O, Cohen GN (1986). Interactions of the Escherichia coli methionine repressor with the metF operator and with its corepressor, S-adenosylmethionine. J Biol Chem.

[B90] Cooper A, McAlpine A, Stockley PG (1994). Calorimetric studies of the energetics of protein-DNA interactions in the E. coli methionine repressor (MetJ) system. FEBS Lett.

[B91] Rafferty JB, Somers WS, Saint-Girons I, Phillips SE (1989). Three-dimensional crystal structures of Escherichia coli met repressor with and without corepressor. Nature.

[B92] Somers WS, Phillips SE (1992). Crystal structure of the met repressor-operator complex at 2.8 A resolution reveals DNA recognition by beta-strands. Nature.

[B93] Phillips K, Phillips SE (1994). Electrostatic activation of Escherichia coli methionine repressor. Structure.

[B94] Parsons ID, Persson B, Mekhalfia A, Blackburn GM, Stockley PG (1995). Probing the molecular mechanism of action of co-repressor in the E. coli methionine repressor-operator complex using surface plasmon resonance (SPR). Nucleic Acids Res.

[B95] Aravind L, Koonin EV (1999). DNA-binding proteins and evolution of transcription regulation in the archaea. Nucleic Acids Res.

[B96] Scott JW, Hawley SA, Green KA, Anis M, Stewart G, Scullion GA, Norman DG, Hardie DG (2004). CBS domains form energy-sensing modules whose binding of adenosine ligands is disrupted by disease mutations.. J Clin Invest.

[B97] Kemp BE (2004). Bateman domains and adenosine derivatives form a binding contract.. J Clin Invest.

[B98] Banerjee R, Zou CG (2005). Redox regulation and reaction mechanism of human cystathionine-beta-synthase: a PLP-dependent hemesensor protein. Arch Biochem Biophys.

[B99] McLean JE, Hamaguchi N, Belenky P, Mortimer SE, Stanton M, Hedstrom L (2004). Inosine 5'-monophosphate dehydrogenase binds nucleic acids in vitro and in vivo.. Biochem J.

[B100] Koonin EV, Wolf YI, Karev GP (2002). The structure of the protein universe and genome evolution. Nature.

[B101] Stroupe ME, Leech HK, Daniels DS, Warren MJ, Getzoff ED (2003). CysG structure reveals tetrapyrrole-binding features and novel regulation of siroheme biosynthesis. Nat Struct Biol.

[B102] Vevodova J, Graham RM, Raux E, Schubert HL, Roper DI, Brindley AA, Ian Scott A, Roessner CA, Stamford NP, Stroupe ME, Getzoff ED, Warren MJ, Wilson KS (2004). Structure/function studies on a S-adenosyl-L-methionine-dependent uroporphyrinogen III C methyltransferase (SUMT), a key regulatory enzyme of tetrapyrrole biosynthesis. J Mol Biol.

[B103] Dixon MM, Huang S, Matthews RG, Ludwig M (1996). The structure of the C-terminal domain of methionine synthase: presenting S-adenosylmethionine for reductive methylation of B12. Structure.

[B104] Bandarian V, Pattridge KA, Lennon BW, Huddler DP, Matthews RG, Ludwig ML (2002). Domain alternation switches B(12)-dependent methionine synthase to the activation conformation. Nat Struct Biol.

[B105] Jarrett JT, Huang S, Matthews RG (1998). Methionine synthase exists in two distinct conformations that differ in reactivity toward methyltetrahydrofolate, adenosylmethionine, and flavodoxin. Biochemistry.

[B106] England JL, Shakhnovich BE, Shakhnovich EI (2003). Natural selection of more designable folds: a mechanism for thermophilic adaptation. Proc Natl Acad Sci U S A.

[B107] Murzin AG, Brenner SE, Hubbard T, Chothia C (1995). SCOP: a structural classification of proteins database for the investigation of sequences and structures. J Mol Biol.

[B108] Teichmann SA, Park J, Chothia C (1998). Structural assignments to the Mycoplasma genitalium proteins show extensive gene duplications and domain rearrangements. Proc Natl Acad Sci U S A.

[B109] Gough J, Karplus K, Hughey R, Chothia C (2001). Assignment of homology to genome sequences using a library of hidden Markov models that represent all proteins of known structure. J Mol Biol.

[B110] Madera M, Vogel C, Kummerfeld SK, Chothia C, Gough J (2004). The SUPERFAMILY database in 2004: additions and improvements. Nucleic Acids Res.

[B111] Muller A, MacCallum RM, Sternberg MJ (2002). Structural characterization of the human proteome. Genome Res.

[B112] Keller JP, Smith PM, Benach J, Christendat D, deTitta GT, Hunt JF (2002). The crystal structure of MT0146/CbiT suggests that the putative precorrin-8w decarboxylase is a methyltransferase. Structure (Camb).

[B113] Min J, Feng Q, Li Z, Zhang Y, Xu RM (2003). Structure of the catalytic domain of human DOT1L, a non-SET domain nucleosomal histone methyltransferase.. Cell.

[B114] Christian T, Evilia C, Williams S, Hou YM (2004). Distinct origins of tRNA(m1G37) methyltransferase. J Mol Biol.

[B115] Perez-Rueda E, Collado-Vides J, Segovia L (2004). Phylogenetic distribution of DNA-binding transcription factors in bacteria and archaea. Comput Biol Chem.

[B116] Tatusov RL, Fedorova ND, Jackson JD, Jacobs AR, Kiryutin B, Koonin EV, Krylov DM, Mazumder R, Mekhedov SL, Nikolskaya AN, Rao BS, Smirnov S, Sverdlov AV, Vasudevan S, Wolf YI, Yin JJ, Natale DA (2003). The COG database: an updated version includes eukaryotes. BMC Bioinformatics.

[B117] Anantharaman V, Aravind L, Koonin EV (2003). Emergence of diverse biochemical activities in evolutionarily conserved structural scaffolds of proteins. Curr Opin Chem Biol.

[B118] Mushegian A (2005). Protein content of minimal and ancestral ribosome. RNA.

[B119] Copley RR, Bork P (2000). Homology among (betaalpha)(8) barrels: implications for the evolution of metabolic pathways. J Mol Biol.

[B120] Walker DR, Koonin EV (1997). SEALS: a system for easy analysis of lots of sequences.. Proc Int Conf Intell Syst Mol Biol.

[B121] Altschul SF, Madden TL, Schaffer AA, Zhang J, Zhang Z, Miller W, Lipman DJ (1997). Gapped BLAST and PSI-BLAST: a new generation of protein database search programs.. Nucleic Acids Res.

[B122] Eddy SR (1998). Profile hidden Markov models.. Bioinformatics.

[B123] Sadreyev R, Grishin N (2003). COMPASS: a tool for comparison of multiple protein alignments with assessment of statistical significance.. J Mol Biol.

[B124] Guda C, Lu S, Scheeff ED, Bourne PE, Shindyalov IN (2004). CE-MC: a multiple protein structure alignment server.. Nucleic Acids Res.

[B125] Holm L, Sander C (1994). Searching protein structure databases has come of age.. Proteins.

[B126] Edgar RC (2004). MUSCLE: multiple sequence alignment with high accuracy and high throughput.. Nucleic Acids Res.

[B127] DeLano WL (2002). The PyMOL Molecular Graphics System.

[B128] McGuffin LJ, Bryson K, Jones DT (2000). The PSIPRED protein structure prediction server. Bioinformatics.

[B129] Rost B, Yachdav G, Liu J (2004). The PredictProtein server. Nucleic Acids Res.

[B130] Karplus K, Karchin R, Draper J, Casper J, Mandel-Gutfreund Y, Diekhans M, Hughey R (2003). Combining local-structure, fold-recognition, and new fold methods for protein structure prediction. Proteins.

[B131] Rychlewski L, Jaroszewski L, Li W, Godzik A (2000). Comparison of sequence profiles. Strategies for structural predictions using sequence information. Protein Sci.

[B132] Kelley LA, MacCallum RM, Sternberg MJ (2000). Enhanced genome annotation using structural profiles in the program 3D-PSSM. J Mol Biol.

[B133] Fischer D (2000). Hybrid fold recognition: combining sequence derived properties with evolutionary information. Pac Symp Biocomput.

[B134] Ginalski K, Pas J, Wyrwicz LS, von Grotthuss M, Bujnicki JM, Rychlewski L (2003). ORFeus: Detection of distant homology using sequence profiles and predicted secondary structure. Nucleic Acids Res.

[B135] Shi J, Blundell TL, Mizuguchi K (2001). FUGUE: sequence-structure homology recognition using environment-specific substitution tables and structure-dependent gap penalties. J Mol Biol.

[B136] Lundstrom J, Rychlewski L, Bujnicki J, Elofsson A (2001). Pcons: a neural-network-based consensus predictor that improves fold recognition. Protein Sci.

[B137] Michalopoulos I, Torrance GM, Gilbert DR, Westhead DR (2004). TOPS: an enhanced database of protein structural topology. Nucleic Acids Res.

[B138] Mirkin BG, Fenner TI, Galperin MY, Koonin EV (2003). Algorithms for computing parsimonious evolutionary scenarios for genome evolution, the last universal common ancestor and dominance of horizontal gene transfer in the evolution of prokaryotes. BMC Evol Biol.

[B139] SCOP Superfamily: S-adenosyl-L-methionine-dependent methyltransferases. [http://scop.mrc-lmb.cam.ac.uk/scop/searchcgi?sunid=53335].

[B140] UniProt Knowledgebase: list of organism identification codes. [http://www.expasy.org/cgi-bin/speclist].

[B141] Marchler-Bauer A, Anderson JB, DeWeese-Scott C, Fedorova ND, Geer LY, He S, Hurwitz DI, Jackson JD, Jacobs AR, Lanczycki CJ, Liebert CA, Liu C, Madej T, Marchler GH, Mazumder R, Nikolskaya AN, Panchenko AR, Rao BS, Shoemaker BA, Simonyan V, Song JS, Thiessen PA, Vasudevan S, Wang Y, Yamashita RA, Yin JJ, Bryant SH (2003). CDD: a curated Entrez database of conserved domain alignments.. Nucleic Acids Res.

[B142] Imai A, Matsuyama T, Hanzawa Y, Akiyama T, Tamaoki M, Saji H, Shirano Y, Kato T, Hayashi H, Shibata D, Tabata S, Komeda Y, Takahashi T (2004). Spermidine synthase genes are essential for survival of Arabidopsis. Plant Physiol.

[B143] Imai A, Akiyama T, Kato T, Sato S, Tabata S, Yamamoto KT, Takahashi T (2004). Spermine is not essential for survival of Arabidopsis. FEBS Lett.

[B144] Chattopadhyay MK, Tabor CW, Tabor H (2003). Spermidine but not spermine is essential for hypusine biosynthesis and growth in Saccharomyces cerevisiae: spermine is converted to spermidine in vivo by the FMS1-amine oxidase. Proc Natl Acad Sci U S A.

[B145] Sapperstein S, Berkower C, Michaelis S (1994). Nucleotide sequence of the yeast STE14 gene, which encodes farnesylcysteine carboxyl methyltransferase, and demonstration of its essential role in a-factor export. Mol Cell Biol.

[B146] Gomez-Gomez L, Carrasco P (1998). Differential expression of the S-adenosyl-L-methionine synthase genes during pea development. Plant Physiol.

[B147] Sanchez-Aguayo I, Rodriguez-Galan JM, Garcia R, Torreblanca J, Pardo JM (2004). Salt stress enhances xylem development and expression of S-adenosyl-L-methionine synthase in lignifying tissues of tomato plants. Planta.

[B148] Jarrett JT (2003). The generation of 5'-deoxyadenosyl radicals by adenosylmethionine-dependent radical enzymes. Curr Opin Chem Biol.

